# Quantitative analysis of DNA methylation at all human imprinted regions reveals preservation of epigenetic stability in adult somatic tissue

**DOI:** 10.1186/1756-8935-4-1

**Published:** 2011-01-31

**Authors:** Kathryn Woodfine, Joanna E Huddleston, Adele Murrell

**Affiliations:** 1 Department of Oncology, University of Cambridge, Cancer Research UK Cambridge Research Institute, Cambridge, UK

## Abstract

**Background:**

Genes subject to genomic imprinting are mono-allelically expressed in a parent-of-origin dependent manner. Each imprinted locus has at least one differentially methylated region (DMR) which has allele specific DNA methylation and contributes to imprinted gene expression. Once DMRs are established, they are potentially able to withstand normal genome reprogramming events that occur during cell differentiation and germ-line DMRs are stably maintained throughout development. These DMRs, in addition to being either maternally or paternally methylated, have differences in whether methylation was acquired in the germ-line or post fertilization and are present in a variety of genomic locations with different Cytosine-phosphate guanine (CpG) densities and CTCF binding capacities. We therefore examined the stability of maintenance of DNA methylation imprints and determined the normal baseline DNA methylation levels in several adult tissues for all imprinted genes. In order to do this, we first developed and validated 50 highly specific, quantitative DNA methylation pyrosequencing assays for the known DMRs associated with human imprinted genes.

**Results:**

Remarkable stability of the DNA methylation imprint was observed in all germ-line DMRs and paternally methylated somatic DMRs (which maintained average methylation levels of between 35% - 65% in all somatic tissues, independent of gene expression). Maternally methylated somatic DMRs were found to have more variation with tissue specific methylation patterns. Most DMRs, however, showed some intra-individual variability for DNA methylation levels in peripheral blood, suggesting that more than one DMR needs to be examined in order to get an overall impression of the epigenetic stability in a tissue. The plasticity of DNA methylation at imprinted genes was examined in a panel of normal and cancer cell lines. All cell lines showed changes in DNA methylation, especially at the paternal germ-line and the somatic DMRs.

**Conclusions:**

Our validated pyrosequencing methylation assays can be widely used as a tool to investigate DNA methylation levels of imprinted genes in clinical samples. This first comprehensive analysis of normal methylation levels in adult somatic tissues at human imprinted regions confirm that, despite intra-individual variability and tissue specific expression, imprinted genes faithfully maintain their DNA methylation in healthy adult tissue. DNA methylation levels of a selection of imprinted genes are, therefore, a valuable indicator for epigenetic stability.

## Background

DNA methylation levels at gene promoters and Cytosine-phosphate guanine (CpG) islands associated with gene regulatory regions undergo dynamic changes during differentiation and can vary between normal tissues [1]. In cancer cells epigenetic programming results in global methylation changes [2] and it is difficult to ascertain which methylation changes are abnormal without knowing what normal baseline methylation profiles are for the tissue from which the cancer originates [3]. Since aberrant DNA methylation is thought to be an early indicator of cancer, it will be useful to have a series of reporter loci to indicate the epigenetic health of a tissue sample.

Imprinted genes exhibit monoallelic parent-of-origin specific gene expression. They have roles in fetal growth and development [4] and are usually located within the genome in clusters [5] or as pairs of retrogenes [6]. At present, 64 human genes are known to be subject to genomic imprinting [7] and a further seven show some evidence of imprinted expression. Every imprinted cluster has at least one differentially methylated region (DMR), where DNA methylation is present on one parental allele. A single DMR can regulate a number of imprinted genes within a cluster and, therefore, the methylation status of one DMR can provide information about a number of genes [5]. DMRs can be sub-classified into germ-line and somatic DMRs. Germ-line DMRs are loci which exhibit differences in methylation states between the sperm and the egg. These differences are maintained post-fertilization. At somatic DMRs, DNA methylation is still parent-of-origin specific, but is acquired after fertilization. Once established, DNA methylation imprints are able to withstand genome-wide DNA methylation reprogramming events during the peri-implantation period after fertilization and also during tissue differentiation. Imprinted genes only succumb to genome-wide reprogramming in the primordial germ-line, prior to the resetting of the imprint according to the gonadal sex of the germ-line. This robust feature of maintaining DNA methylation in somatic tissue makes imprinted loci ideal indicators of the overall epigenetic health of a cell.

Many imprinted genes are themselves oncogenes or tumour suppressors [8]; their aberrant expression could drive tumourigenesis. Examples of potential oncogenic imprinted genes include paternally expressed *IGF2, DLK1, PEG1/MEST, PEG3 *and *PEG10 *which are normally expressed in early fetal kidney development and up regulated in Wilms' tumour [9,10]. Aberrant *IGF2 *and *DLK1 *expression has also been shown in adult renal cancers [11]. The down-regulation of the maternally expressed tumour suppressing non-coding *H19 *RNA may leads to cancer in Wilms' tumour and many adult onset cancers [12]. Additionally, the retinoblastoma gene (*RB1*) has recently been shown to have preferential maternal expression [13], thus adding another tumour suppressor to the repertoire of maternally expressed growth inhibiting genes. These examples illustrate that DNA methylation at imprinted regions may have functional roles in oncogenesis and could be used as a surrogate biomarker for loss of imprinting as previously proposed [14] or simply as an indicator of cancer [15].

The best characterised DMR binding protein is CTCF, an 11-zinc finger protein that binds to the *H19*-DMR [16]. CTCF binds the unmethylated maternal allele and mediates the insulator function that prevents the paternal *IGF2 *allele from accessing enhancers downstream of *H19*. It has been shown that mutations of CTCF binding sites within the *H19*-DMR lead to a gain of methylation on the maternal allele, suggesting that CTCF also protects against *de novo *methylation [17-19]. We have recently shown that in Beckwith-Wiedemann and Silver-Russell patients methylation changes at the *H19 *DMR result in concordant changes at the DMRs within the *IGF2 *locus [20]. These changes suggest cross talk between the DMRs in cis, which may be mediated by CTCF, and cohesin, through higher order chromatin looping at the *IGF2/H19 *locus [21,22].

Indications of gene imprinting networks [23] and the identification of protein factors such as ZFP57, a KRAB zinc finger protein that is important for establishing maternal imprints in the oocyte and maintaining methylation at maternal and paternal imprinted domains post-fertilization [24,25], have created the need to analyse larger numbers of imprinted genes in imprinting defects. This may provide more mechanistic clues as to the role of loss of imprinting in cancer and congenital disease than when studying single imprinted genes in isolation.

In order to understand the role that changes in methylation at imprinted genes have in pathophysiology, the normal methylation levels in a variety of tissues and the inter-individual variability needs to be known. High-throughput methylation studies currently employ several technologies but none of these are able to quantitatively identify methylation changes at imprinted genes. This is because imprinted genes often have lower methylation densities at their DMRs (especially the paternally methylated germ-line DMRs [26]) than non-imprinted genes and, therefore, may not be quantitatively detected with antibodies to methyl-CpGs. Many arrays are designed for promoter regions and the DMRs for many imprinted genes are not covered by these arrays. More importantly, however, these technologies rely on the binary detection of either 'methylated' or 'unmethylated' and allele specific methylation associated with imprinted genes means crucial information is lost in such genome wide studies. More focussed methylation studies for imprinted loci in humans are required. One recent approach utilized a microarray representing characterized murine imprinted loci to highlight the tissue specific variability of DMRs [27]. Pyrosequencing (PSQ) is preferable to other methods of methylation analysis in that it is processive and quantitative [28] and reviewed in [29]. PSQ is particularly suited for the examination of selected regions in large numbers of samples [15,30] and avoids some of the pitfalls when using methodologies not tailored to analysing specific CpGs [31].

We have, therefore, designed PSQ assays to cover all the known and potential DMRs of all human imprinted genes identified to date. The methylation levels in eight different tissue types were analysed, representing the widest spectrum of adult human tissues ever assayed for imprinted methylation. A sub-set of DMRs were also assayed in 50 different human blood samples, to establish the intra-individual variation. Our data represents the first comprehensive tissue wide comparison of differential methylation in the human.

## Results

### Novel methylation assays for differentially methylated regions

We designed and optimized 50 PSQ methylation assays to represent all known human imprinted loci, including imprinted clusters, paired imprinted retrogenes and orphan imprinted genes. Our assays provide a quantitative measure of imprinted methylation. Our assays covered 3-9 CpGs within regions either known to, or with the potential to, regulate 51 imprinted genes (Table 1 and Additional File 1; Table S1). We tested our 50 assays on a panel of seven adult tissues [brain, breast, colon, heart, kidney, liver and testis (containing sertoli cells which show imprinted gene expression [32] and sperm cells, which show imprint erasure)] and term placenta. For the purpose of this study, and due to the limitations involved in acquiring high quality human DNA, we have classified the brain as a single tissue, although results from analysis of the mouse brain suggests there may be region dependant parent-of-origin specific expression [33]. The exact location of all assays and the results in each tissue can be downloaded from the laboratory website [34].

**Table 1:** Characteristics of each region investigated

Assay	Chr	Location	P-O-O methylation mark	Germ-line/somatic	Average somatic methylation	Location within gene	CpG island	CTCF	CpG density
DIRAS3 (3)	1	68285444	Maternal	Somatic	70.22	Gene body Exonic	Yes	YV	6.2

DIRAS3 (2)	1	68288969	Maternal	Germ-line#	50.42	Promoter/Ex 1	Yes	N	5.6

DIRAS3 (1)	1	68290053	Maternal	Germ-line#	46.09	5' upstream (GPR)	Yes	YV	5.4

ZDBF2	2	206834066	Paternal	Germ-line	47.65	Intergenic	No	Y	1.4

NAP1L5	4	89837958	Maternal	Germ-line	50.16	Embeded gene promoter	No	N	7.2

ZAC	6	144371274	Maternal	Germ-line	40.83	Isoform promoter	Yes	N	11.2

IGF2R-2	6	160346534	Unknown	Unknown	81.34	Gene body intronic	Yes	N	6.6

SLC22A1	6	160475377	Unknown	Unknown	82.78	Gene body Exonic	Yes	N	5.6

SLC22A3	6	160688909	Unknown	Unknown	39.89	Promoter/Ex 1	Yes	YV	7.6

MEST (s)	7	129913465	Maternal	Somatic	28.15	Isoform promoter	Yes	Y	8.2

MEST	7	129918562	Maternal	Germ-line	45.84	Isoform promoter	Yes	Y	7

GRB10 (g)	7	50817567	Maternal	Germ-line	45.47	Isoform promoter	Yes	YV	12.6

GRB10 (s)	7	50829108	Maternal	Somatic	19.19	Isoform promoter	Yes	YV	6

PEG10	7	94123850	Maternal	Germ-line	55.63	Promoter	Yes	Y	6.4

PON1	7	94791675	Unknown	Unknown	34.04	Promoter/Ex 1	Yes	N	4.6

INPP5FV2	10	121568151	Maternal	Germ-line	60.89	Isoform promoter	Yes	YV	11.2

H19 DMR	11	1977714	Paternal	Germ-line	45.23	**5' upstream (GPR)**	No	Y	5.8

IGF2 (2)	11	2110885	Paternal	Somatic	35.41	Gene body Exonic	Yes	N	6.2

IGF2 (0)	11	2126069	Paternal	Somatic	54.59	Isoform promoter	No	N	2.2

KCNQ1	11	2422247	Unknown	Unknown	22.27	5' upstream (GPR)	Yes	YV	6.8

KvDMR	11	2678628	Maternal	Germ-line	50.68	antisense RNA promoter	Yes	YV	5.6

KCNQ1DN	11	2847182	Unknown	Unknown	26.22	5' upstream (GPR)	Yes	N	11.4

CDKN1C	11	2861764	None	None	12.07	Gene body Exonic	Yes	Y	10.8

OSBPL5 (3)	11	3098345	Unknown	Unknown	82.66	Gene body Exonic	Yes	N	4.2

RB1	13	47791152	Maternal	Germ-line	59.47	Gene body intronic	Yes	N	6.4

DLK	14	100262671	Paternal	Somatic	56.05	Promoter/Ex 1	Yes	N	9.2

IG-DMR	14	100345582	Paternal	Germ-line	58.17	Intergenic	No	N	2.4

MEG3-US	14	100360453	Paternal	Somatic	49.55	Intergenic	Yes	N	6.6

MEG3	14	100361829	Paternal	Somatic	48.99	Promoter/Ex 1	Yes	YV	6.2

RTL	14	100419312	None	None	88.27	Gene body Exonic	Yes	N	6.4

MKRN3	15	21362387	Unknown	Unknown	62.44	Gene body Exonic	No	N	4.4

SNRPN	15	22751911	Maternal	Germ-line	47.12	Isoform promoter	Yes	N	7.8

UBE3A	15	23234499	None	None	3.15	Promoter/Ex 1	Yes	Y	9.6

ATP10A	15	23658720	None	None	13.82	Promoter/Ex 1	Yes	N	5.4

GABRB3	15	24425436	Unknown	Unknown	47.8	Isoform promoter	Yes	YV	7.6

GABRA5	15	24663372	Unknown	Unknown	87.29	Promoter/Ex 1	Yes	YV	13

TCEB3C	18	42797692	None	None	86.52	Embedded gene pronoterd	Yes	YV	7.6

NLRP2	19	60186163	Unknown	Unknown	61.93	Gene-body Exonic	Yes	N	7

PEG3/ZIM2	19	62043527	Maternal	Germ-line	49.92	Gene body intronic	Yes	N	9.2

USP29	19	62322335	Unknown	Unknown	43.27	Intergenic	Yes	N	7.4

ZIM3	19	62348129	Unknown	Unknown	74.21	Promoter/Ex 1	No	N	1

MCTS2	20	21441323	Maternal	Germ-line	44.72	Embedded gene promoter	Yes	Y	6.6

NNAT	20	35582455	Maternal	Germ-line	77.93	Embedded gene promoter	Yes	YV	5.2

BLCAP	20	35589631	None	None	8.37	Promoter/Ex 1	Yes	YV	7.6

L3MBTL	20	41576732	Maternal	Germ-line	51.23	Gene body Exonic (Ex1)	Yes	Y	7.6

NESP	20	56848977	Paternal	Somatic	45.7	Isoform promoter	Yes	Y	9.2

NESPAS	20	56860398	Maternal	Somatic	47.53	Isoform promoter	Yes	N	6.4

GNAS XL	20	56864018	Maternal	Germ-line	43.98	Isoform promoter	Yes	N	5.6

GNAS 1A	20	56898576	Maternal	Somatic	25.1	Isoform promoter	Yes	N	12

Characteristics of each assay: Average somatic methylation is the average level for Brain, Breast, Colon, Heart, Kidney, Liver and Placenta. Genomic localization was analysed by BLASTing the amplicon against UCSC genomic sequence. The location of the amplicon in context of each gene sequence was recorded.

GPR = general promoter region; CpG Island amplicon is within an UCSC annotated CpG island; CTCF binding is determined by analysis of ENCODE tracts on UCSC, (Y = Yes); binding in a tissue specific manner (YV = Yes variable); N = not binding; CpG density was calculated by taking the mid-point of the amplicon used for each assay and analysing the sequence 250bp each side (500bp in total); density is given as No. of CpGs per 100bp; # = the DMR for DIRAS3 could be either DMR1 or DMR2.

Chr = chromosome

The specificity and linearity of the assays were measured using standard templates of known ratios of methylated:unmethylated DNA. The known amount of methylated DNA in the input sample could be compared with the methylation reported by the assay (Additional File 2; Figure S1a). A normal DMR on our standard template contained 50% methylated input DNA and gave an average PSQ read of 50.38%. We therefore defined a DMR as reporting a methylation level by PSQ within the range of 35.73%-65.03% (1.5 times the standard deviation of the mean known DMRs). The assays all report linearity with the increasing amount of methylated DNA in the standard template.

We validated the assays for sensitivity by testing the smallest amount of input DNA required to accurately report levels of 50% methylation in normal samples. As little as 1ng of bisulphite treated DNA could accurately report normal levels of methylation in 23/24 assays. At this low concentration of template, allelic drop-out was only observed in one single assay (*L3MBTL*-DMR). The template DNA of 2.5ng per assay was found to be the lowest amount of DNA required to confidently run a full set of 50 assays that report methylation levels. This is a significant improvement on previous PSQ methodology [28].

The intra-assay reproducibility was assessed by plotting two technical replicates of each assay against one another. The linear relationship between the technical replicates confirmed that intra-assay reproducibility was reliable (regression analysis gave an *r*^2 ^= 0.96, Bland-Altman correlation showed a bias of just 1.60%; Additional File 2, Figure S1b).

Five of our assays were in regions that did not show 35%-65% methylation in any of the tissues tested. Of these *UBE3A, BLCAP *and *CDKN1C *were hypomethylated (average methylation range of 2.96% to 13.53%), while *TCB3C *and *RTL *were hypermethylated (average methylation range of 65.99% to 91.23%). These data are consistent with previous reports that have shown *UBE3A, BLCAP, RTL *and *CDKN1C *to be imprinted genes without promoter DMRs (*UBE3A *[35], *BLCAP *[36], *CDKN1C *[37], *RTL *[38]). All the other assays showed methylation levels consistent with differential methylation in at least one tissue (Additional File 1, Table S2 and Additional File 3, Figure S2). Together, these data represent a catalogue of baseline normal methylation levels in adult tissues.

As the methylation data was analysed on a single platform, we were able to examine the differences of the DMRs when sub-classified into categories such as germ-line or somatic, CpG density and genomic position.

### Germ-line versus somatic DMRs

**Figure 1:**
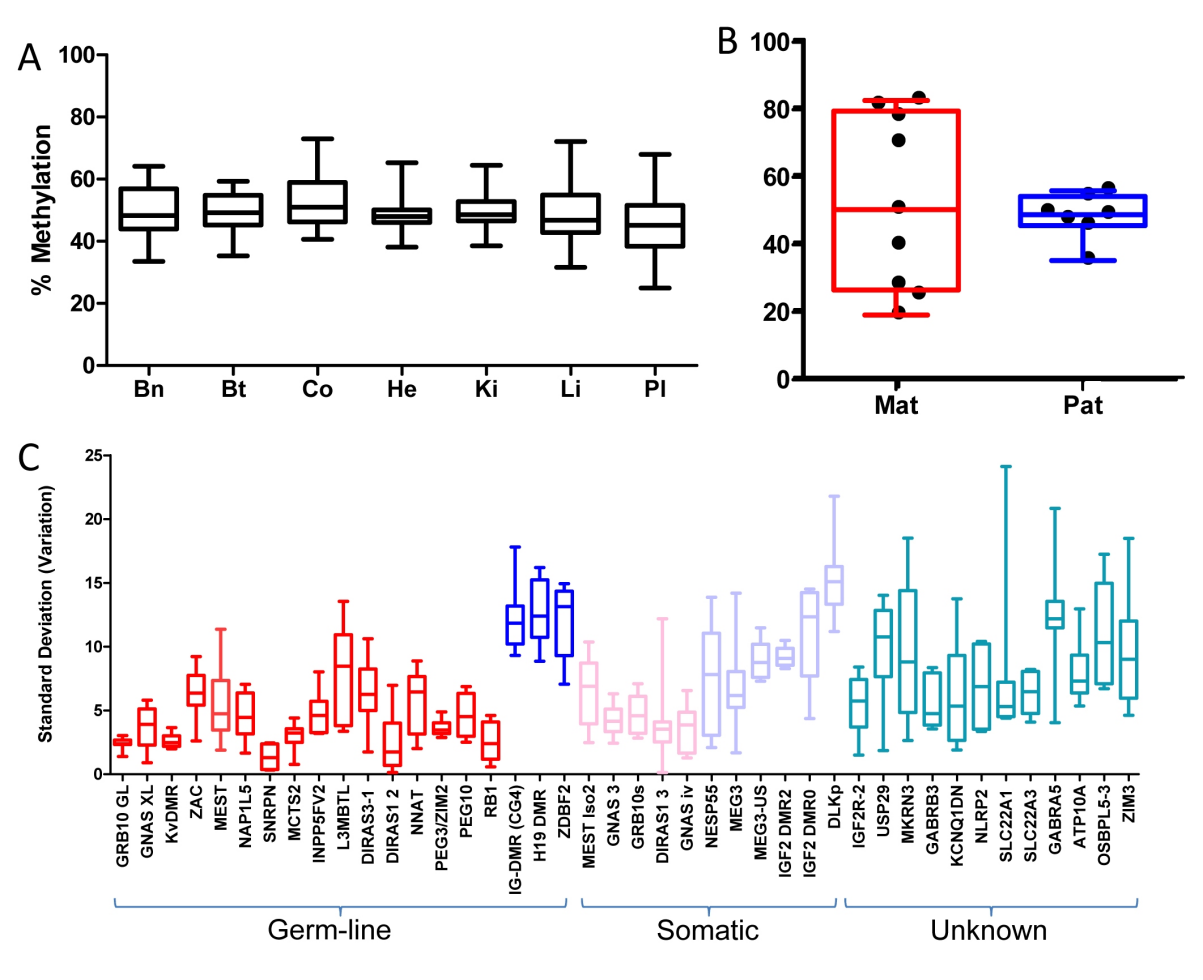
**Average methylation levels for germ-line differentially methylated regions (DMRs; *n *= 17 in each tissue)**. Box plots show median, inter-quartile range and maximum/minimum methylation (Bn = brain; Bt = breast; Co = colon; He = heart; Ki = kidney; Li = liver; Pl = placenta). (b) Average tissue methylation levels of somatic DMRs when analysed by parent-of-origin of the methylation. Each data point represents an individual C-phosphate guanine (CpG). Box plots show median, inter-quartile range and maximum/minimum methylation. (c) Intra-CpG variability of DMR methylation. The standard deviation of all CpGs assayed was calculated for each tissue (*n *= 8). Box plots show median, inter-quartile range and maximum/minimum methylation. Red = maternal germ-line DMRs; pink = maternal somatic DMRs; dark blue = paternal germ-line DMRs; light blue = paternal somatic DMRs; green = parent-of-origin and/or germ-line somatic status unknown.

In order to assess whether germ-line DMRs are more stable than somatic DMRs and would, therefore, have uniform levels of methylation in all tissues, we compared the intra-tissue variability of methylation separately for germ-line and somatic DMRs. The average methylation levels of the 17 germ-line DMRs was 47.26% in all somatic tissues and methylation levels did not vary amongst tissues (Figure 1a). Methylation levels in testis were significantly different from the average methylation of other tissues (matched pair *t*-test *P *= 0.0009): this was expected as the sperm cells in the testis erased the maternal imprints. The germ-line DMRs were hypomethylated in the testis, with the exception of *H19*, *IG-DMR *and *ZBDF2 *which are known to be paternally methylated (Additional File 3, Figure S2 and Additional File 1, Table S2). DMRs that were outliers from the 35%-65% figure calculated above include *NNAT *and *INPP5FV2*. *INPP5FV2 *has been published as a germ-line DMR [39] but we found it to be hypermethylated in colon and liver. Although *NNAT *is also a published germ-line DMR [36], our data shows that in adult tissue only the brain reports methylation levels consistent with a DMR: it is hypermethylated in other tissues. *NNAT *has high expression levels in the brain and low levels of expression in other tissues and, therefore, this may not be a human germ-line DMR (RefExA-[40]).

Analyses of somatic DMRs showed more variation in the average methylation per DMR in different tissues as expected. Many somatic DMRs are only allele specifically methylated in selected tissues - for example, *GNAS 1A *in brain, *MEST *isoform 2 in placenta [41], *MKRN3 *in liver and *SLC22A1 *in placenta - and our data confirms this. We also observed that, for the seven somatic DMRs where the parent of origin is published as paternally methylated, the average methylation levels were between 35%-65% across all tissues, independent of expression and are not tissue specifically variable. In contrast 4/5 maternally methylated somatic DMRs are tissue specific - that is, only reporting methylation levels consistent with a DMR in specific tissues (Figure 1b; Additional File 1, Table S2). However, this does not correlate with expression in adult tissues [40] and imprinted differential methylation is not a good indicator of expression levels in human adult tissue. For example, PON1 reports methylation levels consistent with a DMR in brain and kidney but is only highly expressed in the liver. The somatic DMR at the *MEG *promoter reports levels consistent with a DMR in all tissues but is only expressed in brain, placenta and testis. Tissue specific expression is also often isoform dependent.

As somatic DMRs are thought to be more variable and less stable than germ-line DMRs, we analysed the intra CpG variability of each DMR and compared somatic and germ-line DMRs in all tissues (Figure 1c). The major difference observed was that paternally methylated DMRs showed greater intra-CpG variability across the region assayed than maternally methylated DMRs. Any individual CpG from a maternally methylated DMR is, therefore, more indicative of the rest of the island.

### Effects of CpG density and CTCF binding on variability of differential methylation

We identified which of the DMRs contained CTCF binding sites by matching their genome coordinates for enrichment of CTCF binding in a data set from human liver tissue (M Wilson, unpublished data). We then correlated methylation levels obtained with our assays in liver DNA with CTCF binding within the DMR. Overall, 11/43 DMRs had CTCF binding in liver and, of these, six were germ-line and five were somatic DMRs. There was no evidence of a difference between methylation levels reported in DMRs with CTCF binding sites compared to CTCF binding in the liver (*t*-test, *P *= 0.17; Additional File 4, Figure S3Ai).

In a similar analysis, the average methylation levels reported for all somatic tissues were correlated with published CTCF binding data in four separate cell lines (ENCODE data set [42,43]). Twelve were found to bind CTCF in some cell lines (variable CTCF) and eight bound CTCF in all cell lines. There was also no evidence of difference in methylation levels between DMRs that bound CTCF and those that did not (*t*-test, *P *= 0.99; Additional File 4, Figure S3Aii).

CTCF only binds non-methylated sequences, so we assumed that the somatic DMRs that were hypermethylated in liver (*n *= 10) could still have CTCF binding sites that would be occupied in other tissues but be negative in liver. One surprising result was the CTCF binding to the *GABRA5 *locus in liver, which was 83% methylated. This may be explained by binding of CTCF to a different part of the CpG island to that assayed or it may have been due to the CTCF binding observed being exclusively on the 17% of the GABRA5 that DNA strands within the population that are unmethylated.

Most (38/44) of the regions assayed are CpG islands as defined on the University of California, Santa Cruz (UCSC) genome browser. We also investigated the correlation between CpG density and methylation levels. CpG density was calculated by counting the number of CpG sites 250bp each side of the mid-point of the amplicon used in the assay (Additional File 1, Table S3). The density of CpGs in each amplicon was similar to the CpG density in the overall region CpG island, as annotated by the UCSC genome browser. There was no correlation between the CpG density and average somatic methylation level reported (*r*^2 ^= 0.090; Additional File 4, Figure S3b)

### Effect of genomic position on DMR methylation stability

DMRs can be located intergenically, at promoters or at other regions within the gene body. Additional File 4, Figure S3c, shows no difference between location of the DMR and average somatic methylation levels (ANOVA, *P *= 0.29). The gene body DMRs also most likely have variable methylation levels in somatic tissues. Most of the gene body DMRs were somatic DMRs.

Germ-line DMRs within promoters maintained 35%-65% methylation levels independently of gene expression (for example, *KVDMR, ZAC1, GRB10)*. This was also true for known paternally methylated somatic DMRs. In contrast, known maternal somatic DMRs were hypo/hypermethylated in most tissues and only reported levels consistent with a DMR in one or two tissues. However, this was independent of adult expression levels [40] but may reflect expression during different developmental stages.

### Intra-individual variation of methylation levels at imprinted DMRs

**Figure 2:**
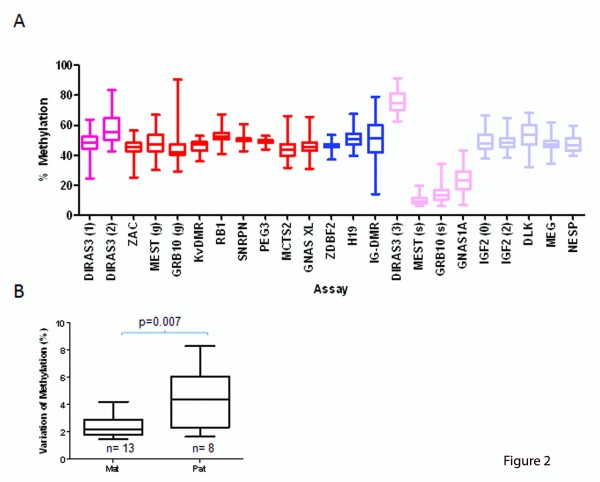
**Analysis of 23 differentially methylated region (DMR) assays in 50 individual blood samples**. (a) Average methylation levels of 50 different individuals. Red = maternal germ-line DMRs; pink = maternal somatic DMRs; dark blue = paternal germ-line DMRs; light blue = paternal somatic DMRs. Box plots show the median, inter-quartile range and maximum/minimum methylation B standard deviation of all C-phosphate guanines (CpGs) assayed for maternal and paternal DMRs. A significant difference was observed between the intra-assay variability reported (matched pair *t*-test, *P *= 0.007).

Methylation levels can vary between individuals due to a number of environmental and genetic reasons. In order to determine the extent of inter-individual variation in methylation levels at imprinted genes in a population, we analysed 23 DMRs in peripheral blood samples from 50 healthy humans. The 23 DMRs chosen represent a range of germ-line and somatic DMRs, incorporating maternally and paternally methylated DMRs. Figure 2a shows the average and range of methylation at each DMR analysed in 50 individuals. Outliers (methylation levels falling outside of the 95% and 99% CI (confidence interval; Additional File 1, Table S4) were observed for 17/23 DMRS (99% CI). However, there were no significant differences in the frequencies of either a germ-line or a somatic DMR being an outlier (*P *= 0.88). This is true for genes that are expressed in the blood (*ZIM2/PEG3, GNAS *and *SNRPN*) as well as those expressed at very low levels (*IGF2, ZAC *and *MEST *[40]).

Nineteen blood samples had outliers for one or more (95% and 99% CI) DMRs. However, no individual is over-represented within the outliers or shows a consistent gain or loss in methylation across several DMRs (repeated measure ANOVA; *P *= 0.60).

Four of the DMRs were consistently either hypermethylated (DIRAS3 - 3) or hypomethylated (*MEST *(s), *GRB10 *(s) and *GNAS1A*) in all samples. These were all maternally methylated DMRs and repeated the observation seen in tissues; paternally methylated somatic DMRs are not tissue specific, whereas somatic maternally methylated DMRs can be tissue specific.

Where DMRs are sequentially present on the same chromosome, loss or gain of methylation at one DMR did not affect the other DMRs *in cis *(comparison of DMRs within the same cluster showed no correlation between methylation levels; max *r*^2 ^= 0.086, data not shown).

Individual CpGs within the maternally methylated DMRS were tightly clustered near the mean methylation level for each individual, whereas the CpGs within the paternally methylated DMRs had a wider range of intra-assay CpG variability. This can be seen for each DMR for all 50 individuals in Additional File 5, Figure S4. This variability for maternal and paternally methylated DMRs was significantly different (Figure 2b, *P *= 0.007). This indicates that in maternal DMRs each CpG changes concurrent with the rest of the island whereas the individual CpGs within paternally methylated DMRs are less likely to be reflective of the rest of the region. This is similar to the trend seen for paternal DMRs which have an increased intra-DMR variability of methylation in blood.

### Detection of methylation changes at Imprinted loci in cell-lines

Cell lines are known to accumulate methylation in passage and are epigenetically unstable, providing a model system with which to examine the plasticity of imprinted DMRs. We examined the methylation levels of nine germ-line DMRs (six maternal and three paternal) and six somatic DMRs (three maternal and three paternal) in a panel of normal and cancer cell lines. Additional File 6, Figure S5 shows a comparison of the methylation levels in cell lines to the baseline levels of methylation as established in blood. All cell lines, even the three cell lines reported to be from normal tissue, had changes in methylation levels. As expected, the cancer cell lines had more variable methylation, some of which could be attributable to karyotype abnormalities (Additional File 1, Table S5 [44]). In this limited sample set we observed that more change was seen in somatic DMRs than in the germ-line DMRs (Additional File 1, Table S5). When evaluated against the tissue specific data (Additional File 3, Figure S2) this was found to be independent from the tissue of origin of the cell line.

**Figure 3:**
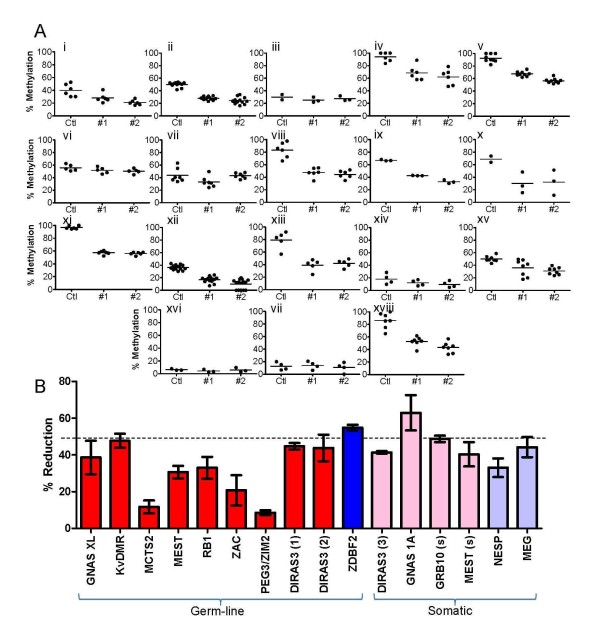
**Methylation changes at differentially methylated regions (DMRs) after treatment with 5 Azacytidine**. (A) Methylation levels of each loci in Sum159 (control) and after treatment with 5 Azacytidine. #1 = 1 nM treatment, #2 = 3 nM treatment. Each data point represents an individual CpG: (i) GNAS XL; (ii) KvDMR; (iii) MCTS2; (iv) MEST (g); (v) RB1; (vi) ZIM2/PEG3; (vii) ZAC; (viii) DIRAS3 (1); (ix) DIRAS3 (2); (x) ZDBF2; (xi) DIRAS3 (3); (xii) GNAS 1A; (xiii) GRB10 (s); (xiv) MEST (s); (xv) NESP55; (xvi) IGF2 (0); (xvii) IGF2 (2); (xviii) MEG3. (b) Percent reduction of methylation at each DMR analysed. Red = maternal germ-line DMRs; pink = maternal somatic DMRs; dark blue = paternal germ-line DMRs; light blue = paternal somatic DMRs. Error bars represent the range of difference between the two experiments.

Sum159 had normal methylation at most DMRs, some assays [*MEST *(g), Retinoblastoma (*RB1), DIRAS3 *(1), *GRB10 *(s) and *MEG3*] reported hypermethylation and hypomethylation was observed at the *IGF2 *assays (Sum159 contains a rearrangement at the RB1 locus [44]). We treated this cell line with 5 Azacytidine (a demetylating agent) to see how the plastic the methylation is at loci reporting normal methylation and to see how reversible the hypermethylation is. A significant decrease in methylation was observed at all loci, except the *IGF2 *DMRs (which already were hypomethylated). Not all DMRs lost methylation at the same rate (Figure 3 and Additional File 1, Table S5). Approximately 30%-60% (average 50%) methylation was lost in 13/18 of the loci (Figure 3a). *ZAC, ZIM2/PEG3 *and *MCTS2 *showed more a modest 10% - 25% reduction in methylation.

## Discussion

Differential DNA methylation has a particular importance in establishing and maintaining mono-allelic imprinted gene expression. Many DMRs are located within defined gene regulatory elements - such as promoters and chromatin insulators. Where the DMR is located within a regulatory element, it is assumed that parent-of-origin specific methylation regulates the binding of transcription factors in an allele specific manner (reviewed in [5]). Most imprinted genes have developmental and tissue restricted expression patterns but, remarkably, many DMRs maintain allele specific methylation in adult somatic tissues independent of absolute levels of expression. In addition, the H19 DMR can function as an ectopic somatic DMR when inserted into a non-imprinted locus. Therefore, the methylation levels and stability of the ectopic DMR is not determined by the parent-of-origin specific marks in the germ-line [45]. When studying cancer, accurate analysis of DNA methylation and the understanding of normal levels are imperative for assessing whether changes in imprinting in complex heterogeneous tissues are physiological or pathophysiological.

We have developed high-throughput, sensitive, quantitative PSQ assays for DNA methylation at all characterized human imprinted loci and have produced the first comprehensive analysis of tissue specific methylation at human imprinted loci. Validation of the PSQ assays proved that the assays were both quantitative and reproducible. The small amount of DNA required and the multiplexing of the assays by using the biotin tag [46,47] means the assays are both efficient and economic for screening large numbers of clinical samples for a variety of different imprinted loci. Recent improvements in ~PSQ technology allows up to 15 different CpG dinucleotides within 80 bp of sequence to be sequentially and accurately assayed for subtle changes in methylation. Therefore, we have meticulously placed the assays within the most suitable part of the DMR and have avoided effects that may be introduced at methylation 'shores' [48]. However, due to the variable CpG content observed within the DMRs it may have been necessary to place an assay in a region with a similar CpG density to these shores.

We analysed known and potential DMR sequences associated with imprinted loci in eight different human adult tissues. We also observed that the methylation levels of most DMRs are maintained in adult somatic tissues and confirmed that germ-line DMRs are particularly stable with no tissue specific gain or loss of methylation. Paternally methylated somatic DMRs are also stable, reporting similar levels across all tissues. Maternally methylated somatic DMRs are more plastic with tissue specific differences observed in adult tissues. This could suggest a difference in the mechanism or developmental stage during which the two types of somatic imprint are established.

We also investigated how methylation may vary within different individuals when the same tissue is studied. For this 23 different DMRs (representative of both maternally and paternally transmitted, germ-line and somatic) in 50 different individual blood samples were analysed. Human samples have varied genetic backgrounds, unlike mice that are inbred onto a limited range of genetic backgrounds. Therefore, it was not surprising that we found locus specific effects in most DMRs, where one or more individuals fell outside of the inter-quartile ranges with maximum or a minimum values bordering on the set thresholds of differential methylation. However, no single individual in our population had disruption of DNA methylation at multiple DMRs, thus excluding an environmental or genetic predisposition to imprinted methylation defects in this sample set.

We noticed that, for paternally methylated DMRs, the methylation at individual CpGs is variable within the DMR. In contrast, maternal DMRs have similar levels of methylation for all CpGs across the regions assayed. This reflects the different make-up of maternal and paternal DMRs, where maternal DMRs are CpG islands, while paternal DMRs do not usually have a CpG density that constitutes the threshold for an island [26]. Our analysis of the effect that CpG density had upon average methylation levels in a DMR showed no specific trends and suggests that, provided enough CpGs are included in an assay, the CpG density at the DMR will not confound the assay.

As our collection of DMRs had a variety of genomic features, we also examined whether normal baseline methylation levels were influenced by CTCF binding sites or genomic position. No significant differences in the average methylation across all tissues were observed between DMRs that contained ubiquitous CTCF binding sites compared to DMRs that contained tissue specific CTCF sites or no CTCF binding in normal tissue. It will be interesting to see whether CTCF affects the ability of DMRs to maintain their unmethylated state in cancer or if changes in CTCF binding lead to changes in gene expression via methylation changes.

Many cell lines have aberrant methylation and, in this study, we found it to be the case for a selection of imprinted genes in cancer cell lines. As methylation is potentially reversible, we examined how effective our assays were in determining quantitative methylation changes after treatment with the demethylating agent, 5 azacytidine. As expected, all methylated DMRs lost methylation after treatment with a demethylation agent but we found that not all loci lost methylation with equal efficiency. While it is uncertain what this means in the context of a single cell line, these results suggest that additional chromatin factors influence the interaction with the maintenance methyltransferases in a locus specific manner.

## Conclusions

Our data provide the first comprehensive catalogue of methylation at imprinted human loci and the level of the variability in methylation in normal samples. In this role, the covalent bond of the methyl group to DNA is important because it is stable and, therefore, the DNA can be easily extracted and investigated in clinical samples. These assays will be valuable in future studies of imprinted regions in disease and in understanding gene regulation at imprinted regions.

## Materials and methods

### DNA samples

Eight different tissue DNAs (from three pooled individuals; brain, breast, colon, heart, kidney, liver, placenta or testis (containing sertoli cells and sperm)) and 50 different blood DNAs were purchased from Amsbio (Abingdon, UK).

### Assay protocol

We designed the assays so they could be easily multiplexed. First, all primers were designed to have melting temperatures of 56°C and to produce a robust polymerase chain reaction (PCR product. We confirmed that the primers were not overlapping annotated single nucleotide polymorphisms (SNPs). Secondly, we opted for two rounds of PCR: the first with gene specific primers (Additional File 1, Table S1), and the second with a common biotinylated primer. The common biotinylated primer both reduced the cost of assay optimization and enabled high throughput second round amplification.

Human genomic DNA was bisulphite converted using the EZ Bisulphite conversion kit (Zymo, CA, USA) following the manufacturer's instructions and eluting in 10 ng/μL (assuming 100% conversion and recovery). DNA was then amplified in a two step amplification using the primers in Additional File 1, Table S1. PCR first round reactions used Thermostart ABGene MasterMix (Fisher, Loughborough, UK), 0.25 μM forward primer, 0.25 μM reverse primer and 10 ng of converted DNA. The assays were amplified in a standard PCR reaction with a melting temperature of 56°C for 25 cycles. This first round PCR was diluted 1:6 and used as a template for a second round PCRs using the same conditions but with 40 cycles and a common biotinylated reverse primer 5' B-CGCCAGGGTTTTCCCAGTCACGAC 3' [46,47]. 10 μL of final PCR product was bound to Streptavidin-sepharose beads (GE Healthcare, Buckinghamshire, UK) and prepared using the Pyromark vacuum tool and buffers (Qiagen, Düsseldorf, Germany) following the manufacturer's protocol. Each sample was run on the Pyromark Q96 machine (Qiagen) using the PyroMark Gold Q96 SQA Reagents (Qiagen). Pyrograms were analysed using the PyroQ CpG software. Graphs were prepared in Graphpad Prism 5.

### Assay validation

In order to validate the assay hypermethylated and hypomethylated DNA (human methylated and non-methylated DNA kit - Zymo Research) was mixed in the following ratios prior to bisulphite treatment as above. DNA was mixed as follows:(1) 100% methylated DNA (M-DNA); (2) 3 M-DNA:1 unmethylated DNA (U-DNA); (3) 2:M-DNA:2:U-DNA; (4) 1M-DNA:3 U-DNA; and (5) 100% U-DNA. This was used as input as described above.

In order to validate the amount of input DNA required, 100 ng, 50 ng, 25 ng and 10 ng of template DNA was used as an input into the Zymo conversion (as above) in final concentrations of 10 ng/μL, 5 ng/μL, 2.5 ng/μL and 1 ng/μL. In order to validate the reproducibility of the assay, duplicate aliquots of 500 ng of template DNA were converted and used in the assays as described above.

### Investigating methylation levels in tissue, blood and cell line samples

1 μg of DNA (tissue samples) or 400 ng (blood) was bisulphite converted using the EZ Bisuphite conversion kit (Zymo) following the manufacturer's instructions and eluting in 10 ng/mL (assuming 100% conversion and recovery). In order to assess DNA levels in different cell lines, DNA was prepared from cells using a DNeasy kit (Qiagen): 500 ng of eluted DNA was bisulphite converted and analysed as described above.

In order to investigate the effect treatment of 5-azacytidine had on the methylation of imprinted regions, Sum159 cells were plated at 40% confluence and treated with 1 nM or 3 nM of 5-azacytidine for 24 h. Cells were then washed three times with phosphate buffered saline and incubated in normal media for a further 24 h. The cells were harvested and DNA extracted with a DNeasy kit (Qiagen). DNA was bisulphite converted and assayed as described above.

### Data analysis

For correlations with genomic features the following data analysis was used.

#### CTCF binding data

Liver specific CTCF binding data was obtained from Mike Wilson (Unpublished data, Duncan Odom Laboratory, Cambridge Research Institute, Cambridge, UK). Briefly, chromatin from prepared from human Liver was chromatin immunoprecipitated with a CTCF antibody and sequenced by Solexa sequencing. CTCF binding sites and DMRs were aligned using Galaxy [49].

Tissue wide CTCF binding data was obtained from UCSC Encode tracks for four different released cell lines (GM12878-lymphoblastoid, K562-leukaemia, NHEK-ectoderm, HUVEC-mesoderm, [42,43]).

#### Statistical analysis

Statistical analysis was performed in Graphpad Prism as indicated in the text. Student *T*-tests were used to determine whether there were statistically significant differences between: (1) Tissue specific methylation levels and the average methylation level reported by all somatic tissues; (2) for individual methylation levels in peripheral blood DNA samples and the average methylation level of all 50 blood samples; (3) for cell line methylation levels compared to average blood methylation levels; and (4 to compare methylation levels of untreated 5-azacytidine treated cell lines matched pair *T *tests were performed. Graphpad Prism was used for all comparison.

## Abbreviations

DMR: differentially methylated region; PCR: polymerase chain reaction; PSQ: pyrosequencing; SNP: single-nucleotide polymorphism.

## Competing interests

The authors declare that they have no competing interests.

## Authors' contributions

KW and AM conceived and designed the study and wrote the manuscript. KW and JH performed the experiments and analysed the data. All authors have read and approved the final manuscript.

## Supplementary Material

Additional file 1 **Supplementary Tables**. *Table S1*: Regions assayed and primer sequences for each pyrosequencing assay. (Top) Actual primers designed to bisulphate treated DNA. (Bottom) Corresponding genomic DNA sequence of primers for location within genome.
*Table S2: *Methylation levels of each region assayed in eight adult tissues. Shaded cells show assays that report levels consistent with a differentially methylated region (DMR). Blue/bold text indicate novel DMRs.
*Table S3: *Characteristics of the differentially methylated region (DMR) assays. Amplicon co-ordinates and genomic locations are shown. CTCF binding sites and C-phosphate guanine (CpG) density are also calculated.
Table S4: Twenty-three differentially methylated regions were analysed in 50 different blood samples.
*Table S5: *Changes of methylation in cell lines and after 5-azacytidine treatment. Click here for file

Additional file 2 ***Figure S2: *Quality control on methylation assays**. (a) Prior to bisulphite treatment, unmethylated and methylated DNA were mixed together in the ratios described. (i) ZAC differentially methylated region (DMR); (ii) GRB10 germ-line DMR; iii: GNAS germ-line DMR; (iv) MCTS2 DMR; (v) KvDMR; (vi) SNRPN DMR. (b) Reproducibility of experiments. The same DNA was independently bisulphite converted and the pyrosquencing assay run. Individual C-phosphate guanines (CpGs) for replicate 1 were plotted against replicate 2. The *r*^2 ^of the correlation was 0.86 and the gradient of the trend-line 0.99. When this is plotted per DMR, *r*^2 ^is 0.96 and *x *= 1.05. Limits of agreement calculated by a Bland-Altman correlation show a difference of 1.60 between the two replicates. This is not significant. Click here for file

Additional file 3 ***Figure S2: *Methylation levels at eight different adult tissues for 50 regions assayed (in chromosome order)**. Bn = brain; Bt = breast; Co = colon; He = heart; Ki = kidney; Li = liver; Pl = placenta; Te = testis; Bl = blood. Each data point represents an individual C-phosphate guanine (CpG). Bars represent the mean methylation level. Click here for file

Additional file 4 ***Figure S3*: Comparison of region characteristics with methylation levels**. (a) Effect of CTCF binding on methylation levels. (i) Average methylation levels of differentially methylated region (DMR) assays in all tissues (CTCF binding determined from Encode Data on UCSC database). (ii) Methylation levels of DMR assays in liver. (b) Correlation between C-phosphate guanine (CpG) densities of each assay with average somatic methylation level reported. Closed circles = germ-line DMRs; open circles = somatic DMRs. (c) Effect of genomic position of DMR on methylation levels. Click here for file

Additional file 5 ***Figure S4: *Methylation levels reported by individual C-phosphate guanines (CpGs) in 50 different individuals at 23 different differentially methylated regions (DMRs**. Red = maternal germ-line DMRs; pink = maternal somatic DMRs; dark blue = paternal germ-line DMRs; light blue = paternal somatic DMRs. Each data point represents an individual CpG. Bars represent the mean methylation levels. A = DIRAS3 (1); B = DIRAS3 (2); C = DIRAS3 (3); D = ZDBF2; E = ZAC; F = MEST (g); G = MEST (s); H = GRB10 (g); I = GRB10 (s); J = H19; K = IGF2-0; L = IGF2-2; M = KvDMR; N = RB1; O = DLK; P = IG-DMR; Q = MEG; R = SNRPN; S = PEG3; T = MCTS2; U = NESP; V = GNAS XL; W = GNAS 1A. Click here for file

Additional file 6 ***Figure S5: *Methylation levels in eight different cell lines and the average blood methylation levels**. Cell lines to the left of the dashed line are normal, whereas cell lines to the right of the line are derived from cancerous samples. Each data point represents an individual C-phosphate guanine (CpG). (a) Maternal germ-line differentially methylated regions (DMRs). (b) Paternal germ-line DMRs. C = maternal somatic DMRs; D = maternal somatic DMRs. Click here for file

## References

[B1] Mohn F Schubeler D Genetics and epigenetics: stability and plasticity during cellular differentiation Trends Genet 2009 25 3 129 136 10.1016/j.tig.2008.12.005 19185382

[B2] Warnecke PM Bestor TH Cytosine methylation and human cancer Curr Opin Oncol 2000 12 1 68 73 10.1097/00001622-200001000-00012 10687732

[B3] Novik KL Nimmrich I Genc B Maier S Piepenbrock C Olek A Beck S Epigenomics: genome-wide study of methylation phenomena Curr Issues Mol Biol 2002 4 4 111 128 12432963

[B4] Reik W Walter J Genomic imprinting: parental influence on the genome Nat Rev Genet 2001 2 1 21 32 10.1038/35047554 11253064

[B5] Edwards CA Ferguson-Smith AC Mechanisms regulating imprinted genes in clusters Curr Opin Cell Biol 2007 19 3 281 289 10.1016/j.ceb.2007.04.013 17467259

[B6] Wood AJ Oakey RJ Genomic imprinting in mammals: emerging themes and established theories PLoS Genet 2006 2 11 e147 10.1371/journal.pgen.0020147 17121465 PMC1657038

[B7] Geneimprint http://www.geneimprint.com

[B8] Murrell A Genomic imprinting and cancer: from primordial germ cells to somatic cells ScientificWorldJournal 2006 6 1888 1910 10.1100/tsw.2006.318 17205195 PMC5917277

[B9] Dekel B Metsuyanim S Schmidt-Ott KM Fridman E Jacob-Hirsch J Simon A Pinthus J Mor Y Barasch J Amariglio N Multiple imprinted and stemness genes provide a link between normal and tumor progenitor cells of the developing human kidney Cancer Res 2006 66 12 6040 6049 10.1158/0008-5472.CAN-05-4528 16778176

[B10] Astuti D Latif F Wagner K Gentle D Cooper WN Catchpoole D Grundy R Ferguson-Smith AC Maher ER Epigenetic alteration at the DLK1-GTL2 imprinted domain in human neoplasia: analysis of neuroblastoma, phaeochromocytoma and Wilms' tumour Br J Cancer 2005 92 8 1574 1580 10.1038/sj.bjc.6602478 15798773 PMC2362015

[B11] Kawakami T Chano T Minami K Okabe H Okada Y Okamoto K Imprinted DLK1 is a putative tumor suppressor gene and inactivated by epimutation at the region upstream of GTL2 in human renal cell carcinoma Hum Mol Genet 2006 15 6 821 830 10.1093/hmg/ddl001 16439445

[B12] Yoshimizu T Miroglio A Ripoche MA Gabory A Vernucci M Riccio A Colnot S Godard C Terris B Jammes H The H19 locus acts *in vivo *as a tumor suppressor Proc Natl Acad Sci USA 2008 105 34 12417 12422 10.1073/pnas.0801540105 18719115 PMC2527926

[B13] Kanber D Berulava T Ammerpohl O Mitter D Richter J Siebert R Horsthemke B Lohmann D Buiting K The human retinoblastoma gene is imprinted PLoS Genet 2009 5 12 e1000790 10.1371/journal.pgen.1000790 20041224 PMC2791201

[B14] Cui H Cruz-Correa M Giardiello FM Hutcheon DF Kafonek DR Brandenburg S Wu Y He X Powe NR Feinberg AP Loss of IGF2 imprinting: a potential marker of colorectal cancer risk Science 2003 299 5613 1753 1755 10.1126/science.1080902 12637750

[B15] Ito Y Koessler T Ibrahim AE Rai S Vowler SL Abu-Amero S Silva AL Maia AT Huddleston JE Uribe-Lewis S Somatically acquired hypomethylation of IGF2 in breast and colorectal cancer Hum Mol Genet 2008 17 17 2633 2643 10.1093/hmg/ddn163 18541649 PMC2515372

[B16] Bell AC Felsenfeld G Methylation of a CTCF-dependent boundary controls imprinted expression of the Igf2 gene Nature 2000 405 6785 482 485 10.1038/35013100 10839546

[B17] Pant V Mariano P Kanduri C Mattsson A Lobanenkov V Heuchel R Ohlsson R The nucleotides responsible for the direct physical contact between the chromatin insulator protein CTCF and the H19 imprinting control region manifest parent of origin-specific long-distance insulation and methylation-free domains Genes Dev 2003 17 5 586 590 10.1101/gad.254903 12629040 PMC196004

[B18] Fedoriw AM Stein P Svoboda P Schultz RM Bartolomei MS Transgenic RNAi reveals essential function for CTCF in H19 gene imprinting Science 2004 303 5655 238 240 10.1126/science.1090934 14716017

[B19] Schoenherr CJ Levorse JM Tilghman SM CTCF maintains differential methylation at the Igf2/H19 locus Nat Genet 2003 33 1 66 69 10.1038/ng1057 12461525

[B20] Murrell A Ito Y Verde G Huddleston J Woodfine K Silengo MC Spreafico F Perotti D De Crescenzo A Sparago A Distinct methylation changes at the IGF2-H19 locus in congenital growth disorders and cancer PLoS One 2008 3 3 e1849 10.1371/journal.pone.0001849 18365005 PMC2268001

[B21] Kurukuti S Tiwari VK Tavoosidana G Pugacheva E Murrell A Zhao Z Lobanenkov V Reik W Ohlsson R CTCF binding at the H19 imprinting control region mediates maternally inherited higher-order chromatin conformation to restrict enhancer access to Igf2 Proc Natl Acad Sci USA 2006 103 28 10684 10689 10.1073/pnas.0600326103 16815976 PMC1484419

[B22] Nativio R Wendt KS Ito Y Huddleston JE Uribe-Lewis S Woodfine K Krueger C Reik W Peters J-M Murrell A Cohesin Is required for higher-order chromatin conformation at the imprinted *IGF2-H19l l*ocus PLoS Genet 2009 5 11 e1000739 10.1371/journal.pgen.1000739 19956766 PMC2776306

[B23] Varrault A Gueydan C Delalbre A Bellmann A Houssami S Aknin C Severac D Chotard L Kahli M Le Digarcher A Zac1 regulates an imprinted gene network critically involved in the control of embryonic growth Dev Cell 2006 11 5 711 722 10.1016/j.devcel.2006.09.003 17084362

[B24] Li X Ito M Zhou F Youngson N Zuo X Leder P Ferguson-Smith AC A maternal-zygotic effect gene, Zfp57, maintains both maternal and paternal imprints Dev Cell 2008 15 4 547 557 10.1016/j.devcel.2008.08.014 18854139 PMC2593089

[B25] Mackay DJ Callaway JL Marks SM White HE Acerini CL Boonen SE Dayanikli P Firth HV Goodship JA Haemers AP Hypomethylation of multiple imprinted loci in individuals with transient neonatal diabetes is associated with mutations in ZFP57 Nat Genet 2008 40 8 949 951 10.1038/ng.187 18622393

[B26] Bourc'his D Bestor TH Origins of extreme sexual dimorphism in genomic imprinting Cytogenet Genome Res 2006 113 1-4 36 40 10.1159/000090813 16575161

[B27] Dindot SV Person R Strivens M Garcia R Beaudet AL Epigenetic profiling at mouse imprinted gene clusters reveals novel epigenetic and genetic features at differentially methylated regions Genome Res 2009 19 8 1374 1383 10.1101/gr.089185.108 19542493 PMC2720189

[B28] Dejeux E El abdalaoui H Gut IG Tost J Identification and quantification of differentially methylated loci by the pyrosequencing technology Methods Mol Biol 2009 507 189 205 full_text 18987816 10.1007/978-1-59745-522-0_15

[B29] Zilberman D Henikoff S Genome-wide analysis of DNA methylation patterns Development 2007 134 22 3959 3965 10.1242/dev.001131 17928417

[B30] Murrell A Rakyan VK Beck S From genome to epigenome Hum Mol Genet 2005 14 Spec No 1 R3 R10 10.1093/hmg/ddi110 15809270

[B31] Nair SS Coolen MW Stirzaker C Song JZ Statham AL Strbenac D Robinson MD Clark SJ Comparison of methyl-DNA immunoprecipitation (MeDIP) and methyl-CpG binding domain (MBD) protein capture for genome-wide DNA methylation analysis reveal CpG sequence coverage bias Epigenetics 2010 6 1 10.4161/epi.6.1.13313 20818161

[B32] Szabo PE Mann JR Biallelic expression of imprinted genes in the mouse germ line: implications for erasure, establishment, and mechanisms of genomic imprinting Genes Dev 1995 9 15 1857 1868 10.1101/gad.9.15.1857 7649473

[B33] Gregg C Zhang J Butler JE Haig D Dulac C Sex-specific parent-of-origin allelic expression in the mouse brain Science 329 5992 682 685 10.1126/science.1190831 20616234 PMC2997643

[B34] Cancer Research UK Epigenetics and imprinting http://www.cambridgecancer.org.uk/research/loc/cambridge/ccri/murrella/?view=CRI&source=research

[B35] Horsthemke B Wagstaff J Mechanisms of imprinting of the Prader-Willi/Angelman region Am J Med Genet A 2008 146A 16 2041 2052 10.1002/ajmg.a.32364 18627066

[B36] Evans HK Wylie AA Murphy SK Jirtle RL The neuronatin gene resides in a 'micro-imprinted' domain on human chromosome 20q11.2 Genomics 2001 77 1-2 99 104 10.1006/geno.2001.6612 11543638

[B37] Monk D Arnaud P Apostolidou S Hills FA Kelsey G Stanier P Feil R Moore GE Limited evolutionary conservation of imprinting in the human placenta Proc Natl Acad Sci USA 2006 103 17 6623 6628 10.1073/pnas.0511031103 16614068 PMC1564202

[B38] Edwards CA Mungall AJ Matthews L Ryder E Gray DJ Pask AJ Shaw G Graves JA Rogers J Dunham I The evolution of the DLK1-DIO3 imprinted domain in mammals PLoS Biol 2008 6 6 e135 10.1371/journal.pbio.0060135 18532878 PMC2408620

[B39] Choi JD Underkoffler LA Wood AJ Collins JN Williams PT Golden JA Schuster EF Jr Loomes KM Oakey RJ A novel variant of Inpp5f is imprinted in brain, and its expression is correlated with differential methylation of an internal CpG island Mol Cell Biol 2005 25 13 5514 5522 10.1128/MCB.25.13.5514-5522.2005 15964807 PMC1156974

[B40] University of Tokyo Laboratory for Systems Biology and Medicine Expression data base http://www.lsbm.org/site_e/index.html 12795199

[B41] McMinn J Wei M Sadovsky Y Thaker HM Tycko B Imprinting of PEG1/MEST isoform 2 in human placenta Placenta 2006 27 2-3 119 126 10.1016/j.placenta.2004.12.003 16338457

[B42] Celniker SE Dillon LA Gerstein MB Gunsalus KC Henikoff S Karpen GH Kellis M Lai EC Lieb JD MacAlpine DM Unlocking the secrets of the genome Nature 2009 459 7249 927 930 10.1038/459927a 19536255 PMC2843545

[B43] Mikkelsen TS Ku M Jaffe DB Issac B Lieberman E Giannoukos G Alvarez P Brockman W Kim TK Koche RP Genome-wide maps of chromatin state in pluripotent and lineage-committed cells Nature 2007 448 7153 553 560 10.1038/nature06008 17603471 PMC2921165

[B44] SKY Karyotypes and FISH analysis of Epithelial Cancer Cell Lines http://www.path.cam.ac.uk/~pawefish/

[B45] Gebert C Kunkel D Grinberg A Pfeifer K H19 imprinting control region methylation requires an imprinted environment only in the male germ line Mol Cell Biol 30 5 1108 1115 10.1128/MCB.00575-09 20038532 PMC2820884

[B46] Guo DC Milewicz DM Universal primer applications for pyrosequencing Methods Mol Biol 2007 373 57 62 17185757 10.1385/1-59745-377-3:57

[B47] Royo JL Hidalgo M Ruiz A Pyrosequencing protocol using a universal biotinylated primer for mutation detection and SNP genotyping Nat Protoc 2007 2 7 1734 1739 10.1038/nprot.2007.244 17641638

[B48] Irizarry RA Ladd-Acosta C Wen B Wu Z Montano C Onyango P Cui H Gabo K Rongione M Webster M The human colon cancer methylome shows similar hypo- and hypermethylation at conserved tissue-specific CpG island shores Nat Genet 2009 41 2 178 186 10.1038/ng.298 19151715 PMC2729128

[B49] Taylor J Schenck I Blankenberg D Nekrutenko A Using galaxy to perform large-scale interactive data analyses Curr Protoc Bioinformatics 2007 Chapter 10 Unit 10 15. 18428782 10.1002/0471250953.bi1005s19 PMC3418382

