# Supplementary material for: Quantitative analysis of DNA methylation at all human imprinted regions reveals preservation of epigenetic stability in adult somatic tissue
Source: Epigenetics Chromatin. 2011 Jan 31;4:1. doi: 10.1186/1756-8935-4-1 (PMC3038880; doi:10.1186/1756-8935-4-1)
Supplement: Additional file 1 — Supplementary Tables. Table S1: Regions assayed and primer sequences for each pyrosequencing assay. (Top) Actual primers designed to bisulphate treated DNA. (Bottom) Corresponding genomic DNA sequence of primers for location within genome. Table S2: Methylation levels of each region assayed in eight adult tissues. Shaded cells show assays that report levels consistent with a differentially methylated region (DMR). Blue/bold text indicate novel DMRs. Table S3: Characteristics of the differentially methylated region (DMR) assays. Amplicon co-ordinates and genomic locations are shown. CTCF binding sites and C-phosphate guanine (CpG) density are also calculated. Table S4: Twenty-three differentially methylated regions were analysed in 50 different blood samples. Table S5: Changes of methylation in cell lines and after 5-azacytidine treatment. [file 1756-8935-4-1-S1.PDF]

Supplementary Table 1:

Assay	Chr.	Location	F primer (to bisulphite converted DNA)	R primer (to bisulphite converted DNA)	S primer (to bisulphite converted DNA)
Bisulphite converted Sequence for primers					
DIRAS3 (3)	1	68285232-68285593	AGTTTTGGTTTTAAGGAATAGAAGT	AACCCAACAACTAACAATAAATATTTTCA	AAGTTTTATAGGAAGATTAGAG
DIRAS3 (2)	1	68288825-68289061	TGGATTAGTTTTTAGATTGTTGTAGATGT	CCCCAAAAACTCACTCCTCC	GGTTAGTTTTTTATAGTTGGT
DIRAS3 (1)	1	68289843-68290062	AACTCCCTCTAACTTCTTTCTCCCTTACCT	GTTTATAGGGTATTGTTGTTAAAATTTGT	CTTTCTCCCTTACCTAT
ZDBF2	2	206833801-206834399	GGTTTTAAATGATTTTGGGTAGTTTG	CCCCTAATAACTTTATCTAACTTCTCT	GATTTTGGGTAGTTTGG
NAP1L5	4	89837528-89838152	AGGGTAGTAATAGGAGGAATTTGGTGTAGT	AAACTCCTCAACCATCTAACCAACC	GGGAGTTTTTTTAGATTT
ZAC	6	144370610-144371540	GGTTGAATGATAAATGGTAGATGT	ACCTTAACTTTACCCCCAC	TGGTAGGAGGAGGTTT
IGF2R-2	6	160346255-160347492	GTAGTTTTTGTTTGGTTTTTGTTTG	CCTATTCACACATAAAATAACCCCT	GAGGGATGTAGTG
SLC22A1	6	160474352-160475794	AGGGATTAGTTTTTTGGAGTGGGG	TATAAAAAACCCCAAACTCCCCTCAT	TTTTTAAAGGTTATGGTG
SLC22A3	6	160688105-160690598	GTTGTTTTTTGGGGAAAGTTGGGT	TCCTTTTAATCCCTACCAACAACCTA	GTAAGGGTTAAGGGTTG
MEST (s)	7	129913054-129914237	TTTTTGGGAATAGGGTGAAGGT	AACCACACCCCCAAAAAAAACTAAT	AGAATTTTTGGTTTTAGG
MEST	7	129917976-129920347	AAGGGGGTTTTGTTTTTTTAATTGTG	AACCCCACAAACTACCCACAAACC	TTGTTGTAAAGGAAATTT
GRB10 (g)	7	50817247-50818365	CTCTCCAAATACTCAAATAAACTC	GGTAGGGGTTTTTGTAGTTTG	CCAAATACTCAAATAAACTCC
GRB10 (s)	7	50828885-50829132	TAGATGGGGTAAATTGAGGTTTAAAGAGGT	CCAATAAAAAAACAAATTAAACCTATCCC	TTGGTTGGTTGTGAGTA
PEG10	7	94122695-94124563	TTGGTTTAGGTGTGGGATTTT	AAACATTCTAAAATACTACTCCATCTC	AGGTGTGGGATTTTATTT
PON1	7	94791706-94791907	ATAGTTTGGATTTAATTTTTTGGGGG	CAAACAAACAAAACCTCCTAACCC	TTATTTTTAAGAGGGTGA
INPP5FV2	10	121567520-121568375	TTTTGTAAAAAGGAGAAAAATTTTGAAAGTTA	ATCCAACAATAATAATAAAATAACCAAACAC	AAATAGTTGTTTGTATTTAATT
H19 DMR	11	1977556-1977893	TATGGGTATTTTTGGAGGTTTTTT	AATCCCAAACCATAACACTAAAAC	GTTATTTGGGAATAGGAT
IGF2 (2)	11	2110410-2111163	GGTTAGGAGGAGGTTGTAGG	CCAAAACAACTTCCCCAAATAC	GGGTGGGTAGAGTAA
IGF2 (0)	11	2125522-2126721	GAATGGTTAGTTTTGAGGGGTTATGGTA	TATTACACCCTAAACCCAAACTCCT	AGTAAAAGTTATTGGATATATAGT
KCNQ1	11	2421671-2422360	GAGTGGTTTAGAGGGAGGGAAAGTA	CTACATCTCCCCTACTTACTCCT	GGGAGGGAAAGTAGA
KvDMR	11	2676987-2678663	AGGGAAGTTTTAGGGTGTGAATTTTTAGAG	CCAAACCACCCACCTAACAAAAAAC	TGGTAATGTTTGGTATTT
KCNQ1DN	11	2846965-2847913	TTGGTTATTTTTTGGAGTTAAATGATTTTT	CACAAAAACCCATTCTTCCTAACTCC	TGATTGTTTTTTGTTG
CDKN1C	11	2861600-2863600	GGGGTTTTTTGGGTTTTAAATTG	CAAAAAACTACCATAAACCAAATAAATAC	AGGGGAGGTTAGTAAAGT
OSBPL5 (3)	11	3098019-3098653	GGGTTTTAGGAATGAGTTTTTTGGTTTTA	CCCACCCTAATCTATACCTAACTCCCC	GGGGTTTTTGTTTTTAAT
RB1	13	47790537-47791958	GGTAGGGTAGTTTTGGAAATGTTTAAG	AACCACAAACCCTTACCC	AGTTTTGGAAATGTTTAAGAT

DLKp	14	100262505-100263352	TTTGTGTTTTTTTGGGTATTTAATTATTG	TTAATACACCTTCCCTCACACTATACAACA	GTTTTTTTGGGTATTTAATTATTGT
IG-DMR	14	100347146-100348387	TTTTATTATTGAATTGGGTTTGTTAGT	ACAATTCCTACTACAAAATTTCAACA	GAATTGGGTTTGTTAGT
MEG3-US	14	100360104-100360793	GGAGTATTTTATTTGAGGTTTGGGGTTTTT	CACCCCCTTTACAACCTATAAAACTTACCA	GGGTTGGTATTGTGTTTA
MEG3	14	100361797-100362462	GTTAGGTAGGGTGAATTTAGGTATAATGTGTG	AACAACCTCTAACTTACATCTAAAAACCAATT	ATGGTTAAGGTGGGGTTG
RTL	14	100417939-100419726	GGTTAGGTTTGAGTATGGGTGTGGTA	CCACCAAAACCACCAAAAATCCAT	TGTTTTTTTAGGGTAATG
MAGEL2	15	21,441,072-21,441,589	TTGGGTTTTGTTAGTTATGAAAGG	ATACTCTACAAAATCCCTTTACCTATAT	TTTTTTGGGTTTTTAGATGT
MKRN3	15	21362239-21362535	AATTATAGGTAAGGAAAGGGAGGATGTAGT	AAACCTACCCCAACCTCAAAAAAAA	AGGGTTTTTATTTGTAGAG
SNRPN	15	22751129-22752147	CCTACCTCCCAACCACTTCCT	GGGAGATTGGGGGGGGAATT	CCCAACCACTTCCTA
UBE3A	15	23234442-23235508	GAGTTTGGTTTTTAGGAGGTTTAAAT	AAACTAATACCAAAAATAAACCCCTAATA	GGTTTTTAGGAGGTTTAAATT
ATP10A	15	23658397-23660111	TTTGGTTTTTAGTAGGTGAGTTGGG	CCAAACTAACTTTTCCTACCTTTCACTTCA	TATTTAGGGTGGAGGGT
GABRB3	15	24425191-24425621	TGGTAGGTGGTTTATATGGGGATA	TAATCAAACCCACCCTCCCAAAAC	TGGGGAGAAGTAGTTTT
GABRA5	15	24663124-24664572	AGATGTTGTTGAGGGTTTTGGAGAAA	AAAAATACATCCTCCTTCTCCTCC	GAGTATTTTTGTAGAGGGT
TCEB3C	18	42802963-42804754	TGTTTGGGAGGTTATGGTTTTAAAAG	CCCAAAATAACCCCAACTAATTCC	TTTTGAGGGGTTTTAGA
NLRP2	19	60185642-60186647	CTCCTACACCCCCAACCTTTCCAAAT	CTCCTACACCCCCAACCTTTCCAAAT	CTACCCCCACAAACCCT
PEG3	19	62041509-62044849	GGTGGTTTTAGGTTAGTTAGAAAGG	ACCAAACTATAAATAATTAATACCTTTT	AGATTTTGTAGTAGTTTTTTAGATT
ZIM2	19	62041610-62044296	GTTTAGTTAGAGAGTGGGTATAGTGG	CCCTACAACAACCCCTCAA	GGTGTTTTTTTGGGGTA
USP29	19	62322152-62322445	TTTTTAGAGGGAGAGATGGAAGTTGG	ACCACAAACCCCCAAATCTTAAAAAA	TGATTGTATTTGGGTTT
ZIM3	19	62347975-62348430	TGTGGTTTGATAGGATTAGGTGGGGT	CAAAACCCAAACATTTAATTTTCAAAAAAA	GGGTTGTTTTTTGATTG
MCTS2	20	29598738-29598953	GGGGTGTTTGGGTGTTTTTAGTTATT	TCCATAAATCCCACAACCCAATATAA	GGGTTTTTAGATGTTTTT
NNAT	20	35581918-35583650	GGGGAGAGGAAGATTGAGGT	ACCCCTTTCAAAAACTTTAACAT	ATTTTTAAGTATATTTTAATTATT
BLCAP	20	35589437-35590083	AGTTTTTAAAGGTGGTTTGTGGTTGA	CCCCCAAAACCCAAACTAAATCC	ATTTTAGGATGGGTTTT
L3MBTL	20	41576625-41577005	TATGAGGAGAAGAGAGGGTTATGGTAT	AAAACCCAACTCAAAACCTAAAAAAC	GGGAGTAGTAATTTTTAGATTTTTT
NESP	20	56847991-56850548	TTTTTTTTGGGGTTTTGGGGTTTTT	AACAAACCTTAAACTATCCCTCCCC	TGGAGTGTTTGGGTATTA
GNAS iv	20	56859925-56860642	GGTTGTAGTGGGGTTAAAGGA	CTCAAAATTTCCCAAATCCTACTATTCT	GTGGGGTTAAAGGAG
GNAS XL	20	56862220-56864834	AGTGGGAGGAGGGGGTTTAGTTAAAG	AACCCCACCAACCTAACCAAAAAAAT	TTTTTAGGGAGAAAAGTG
GNAS 1A	20	56896848-56901334	TGTTGAAGATGGTTATGAAGTTTAAAGTT	TATCCCAATCAACCCAAACCCTACTC	AAGTTTAGGAGGAGGTT
Genomic DNA sequence					
Assay	Chr.	Location	F primer (genomic)	R primer (genomic)	S primer (genomic)
DIRAS3 (3)	1	68285232-68285593	AGCTTTGGCTCCAAGGAACAGAAGC	GAAAATACCTACTGCCAGTTGCTGGGCT	AAGCCCCACAGGAAGATCAGAG

DIRAS3 (2)	1	68288825-68289061	TGGGCCAGCCCCTCACAGCTGG	GGAGGAGTGAGCTCTTGGGG	GGCCAGCCCCTCACAGCTGGT
DIRAS3 (1)	1	68289843-68290062	TCTGGCTTCTTTCTCCCTTGCCT	GCAGGTTTTGACAGCAGTACCCTATGGGC	CTTTCTCCCTTGCCTGT
ZDBF2	2	206833801-206834399	GGTTTTAAATGATTTTGGGCAGTTTG	AGAGAAGTCAGACAAAGCCATTAGGGG	GATTTTGGGCAGTTTGG
NAP1L5	4	89837528-89838152	AGGGTAGCAACAGGAGGAATCTGGTGCAGT	GGCTGGTCAGATGGCTGAGGAGCCC	GGAGCCCTCCTAGACCT
ZAC	6	144370610-144371540	GGCTGAATGACAAATGGCAGATGC	GTGGGGGCAAAGCCAAGGT	TGGCAGGAGGAGGCCC
IGF2R-2	6	160346255-160347492	GCAGTCCCTGCCTGGCCCTTGTCTG	AGGGGTCACCCTATGTGTGAACAGG	GAGGGATGCAGTG
SLC22A1	6	160474352-160475794	AGGGACCAGTCTCTTGGAGTGGGG	ATGAGGGGAGCCTGGGGCTTCCCACA	CTTCCAAAGGCCATGGTG
SLC22A3	6	160688105-160690598	GCTGCCCTCTGGGGAAAGTTGGGT	CAGGCTGCTGGCAGGGACCAAAAGGA	GCAAGGGCCAAGGGCTG
MEST (s)	7	129913054-129914237	TCCCTGGGAACAGGGTGAAGGC	ACTAGCCCTCCTTGGGGGTGTGGCT	AGAACCTCTGGCCTCAGG
MEST	7	129917976-129920347	AAGGGGGCTTTGCTCTCCTAATTGTG	GGCTTGTGGGCAGCCTGTGGGGTT	CTGCTGTAAAGGAAACCT
GRB10 (g)	7	50817247-50818365	CTCTCCAGGTACTCAGGTGGGCTC	CAGGCTGCAGAGGCCCC	CCAGGTACTCAGGTGGGCTCC
GRB10 (s)	7	50828885-50829132	CAGATGGGGCAAACTGAGGCTCAAAGAGGT	GGGACAGGTCCAATTTGTCCTTTCACTGG	CTGGCTGGCTGTGAGCA
PEG10	7	94122695-94124563	CTGGCTCAGGTGTGGGACCCC	GAGATGGAGTAGCACCCCAGAATGTCT	AGGTGTGGGACCCCATCC
PON1	7	94791706-94791907	ACAGCCTGGACCCAACTTTCTGGGGG	GGGCTAGGAGGCTCTGCCTGCCTG	CCATCCCCAAGAGGGTGA
INPP5FV2	10	121567520-121568375	CCCTGCAAAAAGGAGAAAAATCTTGAAAGTCA	GTGCCTGGCCACTCCATTACCACTGCTGGAC	AAACAGCTGCCTGCACCCAACC
H19 DMR	11	1977556-1977893	CATGGGTATTTCTGGAGGCTTCT	GCTTCAGTGTCATGGCCTGGGACTC	GGTCATCTGGGAATAGGAC
IGF2 (2)	11	2110410-2111163	GGTCAGGAGGAGGCTGCAGG	GTATCTGGGGAAGTTGTCCTGG	GGGTGGGTAGAGCAA
IGF2 (0)	11	2125522-2126721	CCTTGAGGGGTCATGGCA	AGGAGTCTGGGTCCAGGGTGCAACA	AGTAAAAGTTATTGGATATATAGT
KCNQ1	11	2421671-2422360	GAGTGGCTCAGAGGGAGGGAAAGTA	AGGAGCAAGCAGGGGAGATGCAG	GGGAGGGAAAGTAGA
KvDMR	11	2676987-2678663	AGGGAAGCCTCAGGGTGTGAACTTCTAGAG	GCCCTTTGCCAGGTGGGTGGCCTGG	TGGCAATGCTTGGCATTC
KCNQ1DN	11	2846965-2847913	CTGGCTATCTCTTGGAGTTAAATGATTTTT	GGAGCCAGGAAGAATGGGCCCCTGTG	TGATTGTTCCCTGTTG
CDKN1C	11	2861600-2863600	GGGCTCTTTGGGCTCTAAACTG	GTACTCACTTGGCTCACCGCAGCCTCTTG	AGGGGCGGTCAGCAAAGC
OSBPL5 (3)	11	3098019-3098653	GGGCCCTAGGAATGAGCCCCTTGGCCCTA	GGGGAGCCAGGCACAGACCAGGGTGGG	GGGGCCTCTGTCTTCAAC
RB1	13	47790537-47791958	GGCAGGGTAGTCTTGGAAATGCCCAAG	AGTCTTGGAAATGCCCAAGAT	GGGCAAGGGTCTGTGGCC
DLKp	14	100262505-100263352	CTTTGTGTTTTCCTGGGTATCTAACCATTG	TGCTGTACAGTGTGAGGGAACGTGTACCAAA	GTTTTCCTGGGTATCTAACCATTGC
IG-DMR	14	100347146-100348387	CCTACCACTGAACTGGGTTTGCCAGT	CTGAAATTCTGCAGTAGGAATTGC	GAACTGGGTTTGCCAGT
MEG3-US	14	100360104-100360793	GGAGCATCCCACCTGAGGCCTGGGGCTCCC	GCAAGCTCCACAGGCTGTAAAGGGGGTGTT	GGGCTGGCACTGTGTCTA
MEG3	14	100361797-100362462	GCCAGGCAGGGTGAATTCAGGCACAATGTGTG	AACTGGTTCCCAGATGTAAGCCAGAGGCTGTC	ATGGCCAAGGTGGGGCTG
RTL	14	100417939-100419726	GGCCAGGTCTGAGTATGGGTGTGGCA	ATGGACTCCTGGTGGTTCTGGTGG	TGCCTCTCTAGGGCAATG

MKRN3	15	21362239-21362535	AATCACAGGCAAGGAAAGGGAGGATGCAGC	CTCCTCCTGAGGCTGGGGCAGGCCT	AGGGCCTCCACCTGCAGAG
SNRPN	15	22751129-22752147	CCTACCTCCCAGCCACTTCCT	AATTCCCCCCCCAGTC	CCCAGCCACTTCCTA
UBE3A	15	23234442-23235508	GAGCCTGGCTCCTAGGAGGCCCAAATC	CACCAGGGGCCTACTTCTGGCACCAGCCC	GGCTCCTAGGAGGCCCAAATCT
ATP10A	15	23658397-23660111	TGCCTGGTCTTCAGCAGGTGAGTTGGG	TGAAGTGAAAGGCAGGAAAAGCCAGCCTGG	CACTCAGGGTGGAGGGC
GABRB3	15	24425191-24425621	TGGTAGGTGGCCCACATGGGGACA	GCCCTGGGAGGGTGGGCCTGATTA	TGGGGAGAAGCAGCTCC
GABRA5	15	24663124-24664572	AGATGCTGTTGAGGGCCCTGGAGAAA	GGAGGAGAAGGAGGATGCATCCTC	GAGCACCTCTGCAGAGGGC
TCEB3C	18	42802963-42804754	TGCCTGGGAGGTCATGGCCTCAAAAG	GGAATCAGCTGGGGCCATTCTGGG	CTCTGAGGGGTCCCAGA
NLRP2	19	60185642-60186647	TCCTGCACCCCGAGCCTTTCCAGAT	TGCTCCTCATTAGCTCAAAGGCAC	TCTGCGCCCACAGGCCCT
ZIM2	19	62041610-62044296	GCCCAGTCCGAGAGTGGGCACAGTGG	CTGAGGGGCTGCTGCAGGGTC	GGTGCCTTTTTGGGGCA
PEG3	19	62043372-62043808	GGCCAGTCAGAAAGG	AAAGGCACCAACCATCCACAGCCTGGC	AGACCCTGCAGCAGCCCCTCAGACC
USP29	19	62322152-62322445	CCCTCAGAGGGAGAGATGGAAGCTGG	TTCCCCAAGACTTGGGGGCTTGTGGC	TGACTGCACCTGGGCTT
ZIM3	19	62347975-62348430	TGTGGCCTGATAGGATCAGGTGGGGC	TCTCCCTGAAAACCAAATGTCTGGGTTTTG	GGGTTGTTTCTTGACTG
MCTS2	20	29598738-29598953	GGGGTGTTTGGGTGCTCCCAGTCACC	TCATATTGGGCTGTGGGACCCATGGA	GGGCTTCCAGATGCTTCT
NNAT	20	35581918-35583650	GGGGAGCGGAAGACTGAGGT	TGCCAAAGCTTCTGAAAGGGGCAC	ATCCCCAAGCATATTCCAACCACTT
BLCAP	20	35589437-35590083	AGTCCTCAAAGGTGGTCTGTGGCTGA	GGACCTAGCCTGGGCCTTGGGGG	ATCCCAGGATGGGTCCT
L3MBTL	20	41576625-41577005	CATGAGGCGAAGAGAGGGCCATGGCAC	GCTCCCCAGGCCCTGAGCTGGGCCC	GGGAGTCGCAATCCTCAGACCCTCC
NESP55	20	56847991-56850548	CCTTCCCTGGGGCTCTGGGGCTCCT	GGGGAGGGACAGCTCAAGGTCTGCC	TGGAGTGCTTGGGCACCA
GNAS iv	20	56859925-56860642	GGCTGTAGTGGGGCTAAAGGA	AGAACAGCAGGACCTGCGAAACTCTGAG	GTGGGGCTAAAGGAG
GNAS XL	20	56862220-56864834	AGTGGGAGGAGGGGGTCCAGCCAAAG	ACTCCCCTGGCTAGGCTGGTGGGGTC	CCTCCAGGGAGAAAAGTG
GNAS 1A	20	56896848-56901334	TGCTGAAGATGGCCATGAAGCTCAAAGCC	GAGTAGGGCCTGGGCTGACTGGGACA	AAGCCCAGGAGGAGGCC

Supplementary Table 2: Average methylation levels reported in each tissue. Shaded cells show assays that report levels consistent with a DMR.

	Brain	Breast	Colon	Heart	Kidney	Liver	Placenta	Testis	Somatic Av.
**Mat GL**									
GRB10(g)	44.89	49.31	48.64	48.72	38.50	42.77	46.32	17.74	45.47
GNAS XL	43.33	35.27	47.33	47.69	46.56	43.67	38.35	13.24	43.98
KvDMR	48.33	54.65	58.65	46.69	40.28	55.47	42.25	10.36	50.68
ZAC	43.42	44.44	40.64	38.12	46.78	31.59	43.90	26.62	40.83
MEST	56.08	49.64	41.03	48.52	46.96	32.82	44.34	27.86	45.84
NAP1L5	53.33	48.84	54.20	47.99	46.64	48.59	51.56	19.04	50.16
SNRPN	45.30	45.11	48.56	47.04	49.57			27.63	47.12
MCTS2	33.50	45.50	46.55	47.69	49.90	45.19	24.98	21.43	44.72
INPP5FV2	57.81	52.49	72.95	50.26	59.72	72.11	51.04	11.01	60.89
L3MBTL	57.61	47.37	52.62	49.97	50.54	49.27	46.43	16.41	51.23
RB1	64.16	57.39	55.22	65.27	64.45	50.33	52.75	23.86	59.47
PEG3/ZIM2	46.83	46.67	55.83	50.21	50.44	49.54		18.66	49.92
PEG 10	55.97		59.22	51.70				16.59	55.63
DIRAS3 (1)	43.95	47.71	54.32	37.17	39.68	53.71	61.83	16.37	46.09
DIRAS3 (2)	54.19	54.85	45.86	53.34	47.50	46.77	30.11	31.60	50.42
NNAT	50.22	86.06	84.56	82.00	81.17	83.57	74.85	16.43	77.93
**Pat GL**									
H19 DMR	44.56	56.49	44.42	41.72	42.64	41.57	45.15	84.23	45.23
IG-DMR (CG4)	61.01	59.31	64.44	43.37	54.50	66.36	67.93	65.90	58.17
ZDBF2	40.87	49.14	50.96	49.38	51.07	44.48	57.34	75.86	47.65
**Mat Som**									
MEST (s)	25.80	29.72	26.49	17.44	55.60	13.86	37.65	17.28	28.15
GNAS 1A	54.34	20.22	18.83	19.24	25.06	12.89	12.54	13.81	25.10
GRB10s	36.41	23.14	12.72	16.55	10.99	15.32	11.47	8.90	19.19
DIRAS3 (3)	62.66	77.69	61.42	65.21	73.35	80.99	51.38	38.29	70.22
NESPAS	43.18	54.55	49.72	46.13	46.51	45.12	45.36	43.79	47.53
**Pat Som**									
IGF2 DMR0	46.48	60.18	53.77	46.82	55.05	65.25	54.98	54.92	54.59
IGF2 DMR2	38.47	37.37	36.40	36.62	31.92	31.71	30.46	75.49	35.41
NESP	58.13	38.49	51.31	47.93	44.62	33.72	42.32	16.51	45.70
MEG3	49.51	44.79	53.55	42.52	51.37	52.21	39.69	12.75	48.99
MEG3-US		61.22	53.07	38.05	47.30	48.12			49.55
DLK	41.49	65.58	67.18	45.14	53.96	62.93	40.29		56.05
**Unknown**									
IGF2R-2	57.42	83.51	88.49	80.26	88.01	90.34	80.91	10.99	81.34
RTL	90.02	91.10	74.99	91.23	91.74	90.51	65.99	24.95	88.27
USP29	42.86	43.42	62.35	37.31	39.20	34.46	36.36	6.13	43.27
MKRN3	27.36	58.62	66.70	77.87	86.99	57.11	19.42	32.08	62.44
GABRB3	44.63	37.82	56.23	48.01	55.35	44.78	21.50	25.70	47.80
KCNQ1DN	9.82	23.52	39.27	29.32	17.28	38.10	51.88	6.13	26.22
PON1	44.61	28.67	39.17	21.37	42.95	27.45	32.54	12.07	34.04
NLRP2	59.31	64.32	64.39	58.47	59.53	65.57		44.84	61.93
SLC22A1	77.59	78.37	91.85	69.00	92.73	87.15	53.16	26.20	82.78
SLC22A3	37.69	53.45	32.10	31.40	47.50	37.23	20.50	32.21	39.89
GABRA5	89.07	89.26	87.87		86.71	83.52	58.83	21.71	87.29
ATP10A	16.85	9.38	14.68	16.10	12.09		40.56		13.82
OSBPL5	84.79	85.44	82.14	85.19	86.09	72.33	68.31	83.46	82.66
ZIM3	54.47	77.09	88.83	91.67	74.68	58.54	83.53	74.58	74.21
TCEB3C	88.23	85.79	84.94	87.08	88.94	84.14	66.67		86.52
BLCAP	6.34	6.11	11.11	5.48	14.13	7.04	7.17		8.37
UBE3A	2.96	3.89	3.30	2.79	2.72	3.26	2.38	13.60	3.15
KCNQ1	19.81	15.60	18.61	41.99	17.20	20.39	16.79		22.27
CDKN1C	10.44	12.36	13.53	6.63	9.05	20.41	5.59		12.07

Supplementary Table 3:

Assay	Chr.	Amplicon Start	Amplicon Finish	Mid-point	Location within gene	CpG island (UCSC thresholds)	CTCF Any (n=4)	CTCF binding in Liver	CpG density (No. in 500bp)	CpG density (%)
DIRAS3 (3)	1	68285331	68285556	68285444	Gene body exonic	Yes	YV	N	31	6.2
DIRAS3 (2)	1	68288898	68289039	68288969	Promoter/Ex 1	Yes	N	N	28	5.6
DIRAS3 (1)	1	68289986	68290120	68290053	5' upstream (general promoter region)	Yes	YV	Y	27	5.4
ZDBF2	2	206833964	206834167	206833051	Intergenic	No	Y	N	7	1.4
NAP1L5	4	89837764	89838152	89837958	Embeded gene promoter	No	N	N	36	7.2
ZAC	6	144371142	144371405	144370576	Isoform promoter	Yes	N	N	56	11.2
IGF2R-2	6	160346392	160346676	160346155	Gene body intronic	Yes	N	N	33	6.6
SLC22A1	6	160475219	160475534	160474697	Gene body exonic	Yes	N	N	28	5.6
SLC22A3	6	160688719	160689099	160688450	Promoter/Ex 1	Yes	YV	Y	38	7.6
MEST (s)	7	129913367	129913563	129913261	Isoform promoter	Yes	Y	Y	41	8.2
MEST	7	129918329	129918795	129917776	Isoform promoter	Yes	Y	Y	35	7
GRB10 (g)	7	50817512	50817622	50817567	Isoform promoter	Yes	YV	N	63	12.6
GRB10 (s)	7	50828983	50829232	50829108	Isoform promoter	Yes	YV	Y	30	6
PEG10	7	94123774	94123925	94123850	Promoter	Yes	Y	Y	32	6.4
PON1	7	94791561	94791789	94791675	Promoter/Ex 1	Yes	N	N	23	4.6
INPP5FV2	10	121567867	121568434	121567558	Isoform promoter	Yes	YV	N	56	11.2
H19 DMR	11	1977559	1977869	1977714	5' upstream (promoter region of H19)	No	Y	Y	29	5.8
IGF2 (2)	11	2110736	2111033	2110885	Gene body exonic	Yes	N	N	31	6.2
IGF2 (0)	11	2125958	2126179	2126069	Isoform promoter	No	N	N	11	2.2
KCNQ1	11	2422139	2422354	2422247	5' upstream (general promoter region)	Yes	YV	N	34	6.8
KvDMR	11	2678501	2678755	2678628	antisense RNA promoter	Yes	YV	N	28	5.6
KCNQ1DN	11	2847092	2847272	2847182	5' upstream (general promoter region)	Yes	N	N	57	11.4
CDKN1C	11	2861703	2861825	2861764	Gene body exonic	Yes	Y	Y	54	10.8

OSBPL5 (3)	11	3098161	3098529	3098345	Gene body exonic	Yes	N	N	21	4.2
RB1	13	47791138	47791165	47791152	Gene body intronic	Yes	N	N	32	6.4
DLK	14	100262573	100262769	100262505	Promoter/Ex 1	Yes	N	N	46	9.2
IG-DMR	14	100345428	100345735	100347422	Intergenic	No	N	N	12	2.4
MEG3-US	14	100360205	100360701	100360277	Intergenic	Yes	N	N	33	6.6
MEG3	14	100361710	100361948	100361797	Promoter/Ex 1	Yes	YV	N	31	6.2
RTL	14	100419190	100419433	100419190	Gene body exonic	Yes	N	N	32	6.4
MKRN3	15	21362239	21362535	21362387	Gene body exonic	No	N	N	22	4.4
SNRPN	15	22751840	22751982	22751911	Isoform promoter	Yes	N	N	39	7.8
UBE3A	15	23234414	23234583	23234499	Promoter/Ex 1	Yes	Y	Y	48	9.6
ATP10A	15	23658536	23658904	23658720	Promoter/Ex 1	Yes	N	N	27	5.4
GABRB3	15	24425227	24425644	24425436	Isoform promoter	Yes	YV	N	38	7.6
GABRA5	15	24663153	24663590	24663372	Promoter/Ex 1	Yes	YV	Y	65	13
TCEB3C	18	42797485	42797898	42797692	Embeded gene pronoter	Yes	YV	N	38	7.6
NLRP2	19	60186017	60186308	60186163	Gene-body Exonic	Yes	N	N	35	7
PEG3/ZIM2	19	62043458	62043596	62043527	Gene body intronic	Yes	N	N	46	9.2
USP29	19	62322198	62322472	62322335	Intergenic	Yes	N	N	37	7.4
ZIM3	19	62347992	62348266	62348129	Promoter/Ex 1	No	N	N	5	1
MCTS2	20	21441164	21441481	21441323	Embeded gene pronoter	Yes	Y	N	33	6.6
NNAT	20	35582268	35582642	35582455	Embeded gene pronoter	Yes	YV	N	26	5.2
BLCAP	20	35589409	35589852	35589631	Promoter/Ex 1	Yes	YV	N	38	7.6
L3MBTL	20	41576598	41576866	41576732	Gene body exonic (Ex1)	Yes	Y	Y	38	7.6
NESP	20	56848826	56849128	56848977	Isoform promoter	Yes	Y	Y	46	9.2
NESPAS	20	56860287	56860509	56860398	Isoform promoter	Yes	N	N	32	6.4
GNAS XL	20	56863875	56864161	56864018	Isoform promoter	Yes	N	N	28	5.6
GNAS 1A	20	56898332	56898820	56898576	Isoform promoter	Yes	N	N	60	12

Characteristics of the DMR assays: Amplicon co-ordinates and genomic locations are shown. Genomic localisation was analysed by BLASTing the amplicon against UCSC genomic sequence. The location of the amplicon in context of each gene sequence was recorded. CTCF binding sites are calculated as described in the methods section. CTCF is determined as binding (Y-Yes), Binding in a tissue specific manner (YV- Yes variable), Not binding (N). CpG density

was calculated by taking the mid-point of the amplicon used for each assay and analysing the sequence 250bp each side (500bp in total). Density is given as no. of CpGs per 100bp.

Supplementary Table 4: Methylation analysis of 23 different DMRs in 50 individual blood samples.

DMR	Chr: Loc	Mat/Pat	GL/S	Average (Mean)	95% CI	Span#	No outside CI (95%)	99% CI	Span#	No outside CI (99%)	Mean outside 35-65
**DIRAS3 (1)**	1	Mat	?	48.39	47.29-49.49	2.20	34%	46.94-49.84	2.90	26%	2%
**DIRAS3 (2)**	1	Mat	? (GL)	58.22	56.55-59.90	3.35	76%	56.02-60.24	4.22	74%	24%
**DIRAS3 (3)**	1	Mat	S	76.14	75.14-77.13	1.99	56%	74.83-77.45	2.62	32%	98%
**ZDBF2**	2	Pat	GL	46.63	44.53-48.73	4.20	0%*	43.84-49.38	5.54	0%*	0%
**ZAC**	6	Mat	GL	45.77	44.97-46.57	1.60	26%	44.72-46.82	2.10	20%	4%
**GRB10 (g)**	7	Mat	GL	45.07	46.19-43.95	2.24	62%	46.55-43.59	2.96	44%	18%
**GRB10 (s)**	7	?	S	13.78	13.00-14.56	1.56	26%	12.75-14.80	2.05	20%	100%
**MEST (g)**	7	Mat	GL	47.99	49.08-46.90	2.18	56%	46.56-49.42	2.86	48%	6%
**MEST (s)**	7	Mat	S	9.59	8.82-10.36	1.54	4%	8.58-10.60	2.02	2%	100%
**H19**	11	Pat	GL	50.62	49.01-52.24	3.23	6%	48.50-52.75	4.21	0%	4%
**IGF2 (0)**	11	Pat	S	48.75	47.01-50.49	3.58	4%	46.46-51.04	4.58	0%*	2%
**IGF2 (2)**	11	Pat	S	48.96	47.95-49.97	2.02	16%	47.63-50.29	2.66	6%	0%
**KvDMR**	11	Mat	GL	45.61	44.99-46.23	1.24	38%	44.80-46.42	1.62	20%	0%
**RB1**	13	Mat	GL	52.89	52.09-53.69	1.60	16%	51.84-53.94	2.20	6%	2%
**MEG**	14	Pat	S	47.71	46.85-48.56	1.71	8%	46.59-48.82	2.23	8%	2%
**DLK**	14	Pat	S	54.6	52.27-56.92	4.65	18%	50.63-56.89	6.26	16%	16%
**IG-DMR**	14	Pat	GL	50.27	47.39-53.15	5.76	24%	46.24-54.54	8.30	8%	18%
**SNRPN**	15	Mat	GL	50.3	49.74-50.87	1.13	10%	49.56-51.04	1.48	4%	0%
**PEG3/ZIM2**	19	AS	?	49.21	48.57-49.86	1.29	2%	48.29-50.01	1.72	0%	0%
**MCTS2**	20	Mat	GL	44.13	43.01-45.26	2.25	20%	42.66-45.61	2.95	12%	8%
**GNAS XL**	20	Mat	GL	45.61	44.91-46.30	1.39	36%	44.69-46.52	1.83	22%	4%
**GNAS 1A**	20	Mat	S	22.79	22.10-23.48	1.38	62%	21.89-23.70	1.81	28%	100%
**NESP55**	20	Pat	S	47.01	46.38-47.65	1.27	60%	46.18-47.85	1.67	26%	0%

*Not enough CpGs within assay to attach real statistical significance to the results #Span-Intra assay-CpG variability (highest reading minus lowest reading (%))

The mean, 95% and 99% confidence intervals were determined for each DMR. Samples outside the norm calculated for each DMR were determined at the two different confidence intervals and calculated. Samples with the mean methylation level outside those calculated for a DMR (See Sup Fig 1) were also calculated.

Supplementary Table 5: Changes of methylation in cell lines and after 5-azacytidine treatment.

	**HB2**	**HS27**	**IMR90**	**JEG3**	**SUM 159**	**CAL51**	**HCT116**	**COLO205**	***5Aza (1)***	***5Aza (2)***
**Karyotype**	46XX	46XY	46XY	Hypertriploid (modal n = 71)	Hyperdiploid normal in most regions	46XX	45X0 Del on Chr16 by karyotype analysis	Hypertriploid	Hyperdiploid normal in most regions	Hyperdiploid normal in most regions
**Mat GL**										
GNAS XL	No Change	No Change	No Change	Hyper (p=0.0016)	No Change	No Change**	Hyper (0.0001)	Hyper (0.0001)	*Decreased (p=0.0075)*	*Decreased (p=0.0038)*
KvDMR	No Change	Hypo (p=0.0003)	No Change	Hypo (p=0.0001)	No Change	Hypo (p=0.0001)	No Change	No Change	*Decreased (p=0.0001)*	*Decreased (p=0.0001)*
ZAC	Hypo (p=0.0001)	No Change	No Change	Hyper (p=0.0001)	No Change	No Change	No Change	No Change	*Decreased (p=0.005)*	*No Change*
PEG3/ZIM2	No Change	No Change	Hypo (p=0.0001)	Hypo (p=0.0001)	Hyper (p=0.0029)	Hyper (p=0.0001)	Hyper (p=0.0015)	No Change	*No Change*	*No Change*
MCTS2	No Change*	No Change*	No Change*	Hypo*	Hypo*	No Change*	Hyper*	No Change*	*Decreased**	*Decreased**
RB1	Hyper (p=0.0003)	Hyper (p=0.0001)	Hyper (p=0.0001)	Hyper (p=0.0001)	Hyper (p=0.0001)	Hyper (p=0.0001)	Hyper (p=0.0001)	No Change	*Decreased (p=0.0001)*	*Decreased (p=0.0001)*
**Pat GL**										
H19 DMR	No Change	No Change	No Change	No Change	No Change	No Change	No Change	No Change	-	-
IG-DMR	No Change*	No Change*	No Change*	-	-	No Change*	-	-	-	-
ZDBF2	No Change*	Hypo*	No Change*	Hyper*	Hyper*	Hyper*	Hyper*	Hyper*	*Decreased**	*Decreased**
**Mat Som**										
MEST (s)	Hypo (0.0084)	Hyper (p=0.009)	No Change	Hyper (p=0.0027)	Hyper (p=0.0068)	Hyper (p=0.0009)	Hyper (p=0.0050)	No Change	*No Change*	*Decreased (p=0.0153)*
DIRAS3 (3)	Hyper (p=0.0002)	Hyper (p=0.0001)	No Change	Hyper (p=0.0001)	Hyper (p=0.0001)	Hyper (p=0.0001)	-	Hyper (p=0.0078)	*Decreased (p=0.0001)*	*Decreased (p=0.0001)*
GNAS 1A	No Change	No Change	Hyper (p=0.0001)	No Change	Hypo (p=0.0001)	Hypo (p=0.0001)	-	-	*Decreased (p=0.0001)*	*Decreased (p=0.0001)*
**Pat Som**										
NESP55	Hypo (p=0.0001)	No Change	No Change	Hyper (p=0.0001)	Hyper (p=0.0003)	Hyper (p=0.0001)	Hyper (p=0.0001)	Hyper (p=0.0001)	*Decreased (p=0.0081)*	*Decreased (p=0.0001)*
IGF2 (0)	Hypo*	No Change*	No Change*	Hypo*	Hypo*	-	Hypo*	Hypo*	*No Change**	*No Change**
IGF2 (2)	No Change*	No Change	Hypo (p=0.007)	Hyper (p=0.0001)	Hypo (p=0.0001)	Hypo (p=0.0001)	Hyper (p=0.0003)	No Change	*No Change*	*No Change*

*Not enough CpGs to perform matched pair *t*-Test ** *t* test shows no significant difference between the means, but the CpGs show individual changes.

t tests were performed to assess the significance of the change. Cell lines were compared to the average methylation levels in blood. 5-azacytidine treatment was compared to control untreated cells. 99% confidence intervals were used to determine change.
