# Supplementary figures and images for: Quantitative analysis of DNA methylation at all human imprinted regions reveals preservation of epigenetic stability in adult somatic tissue
Source: Epigenetics Chromatin. 2011 Jan 31;4:1. doi: 10.1186/1756-8935-4-1 (PMC3038880; doi:10.1186/1756-8935-4-1)

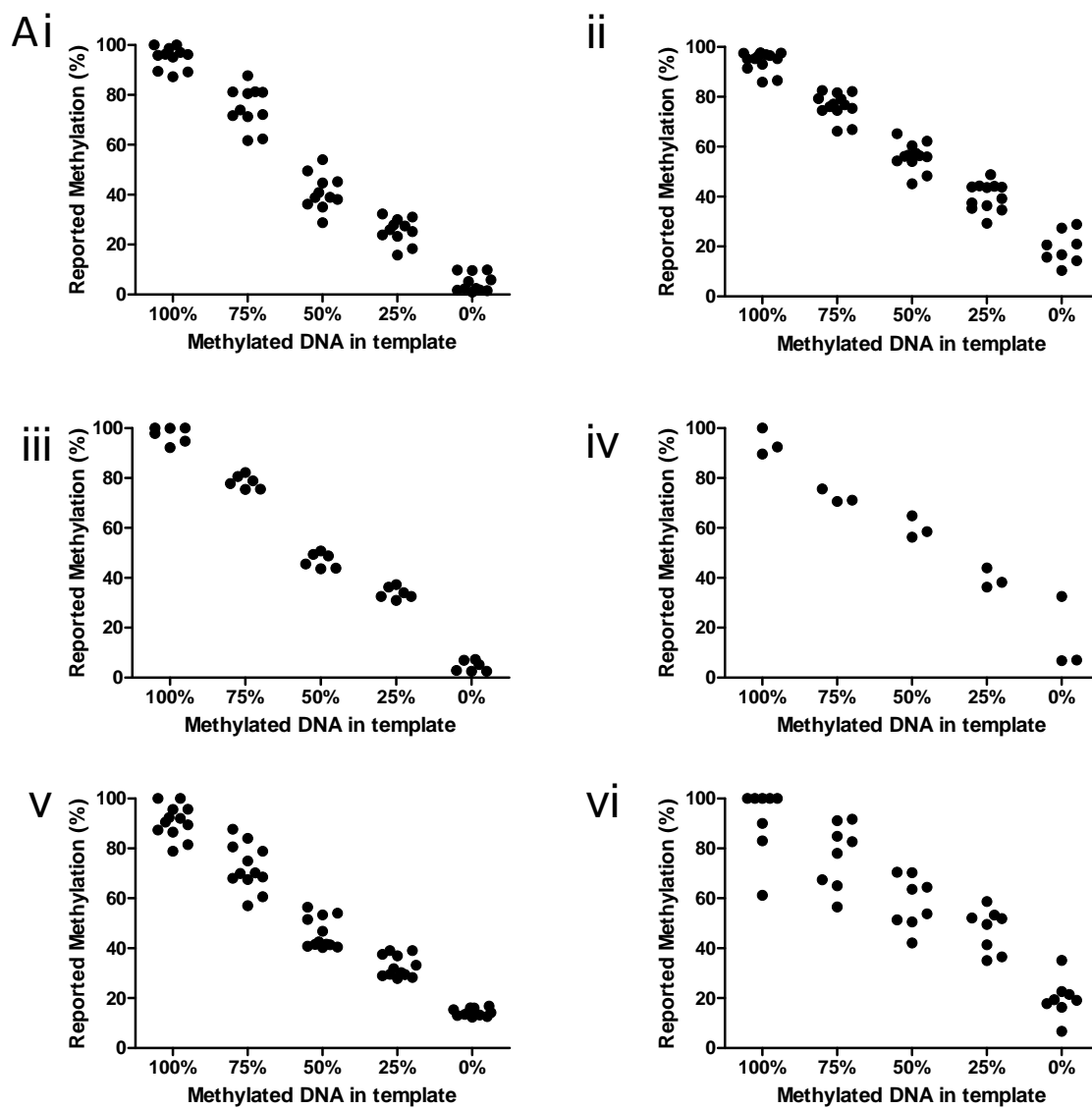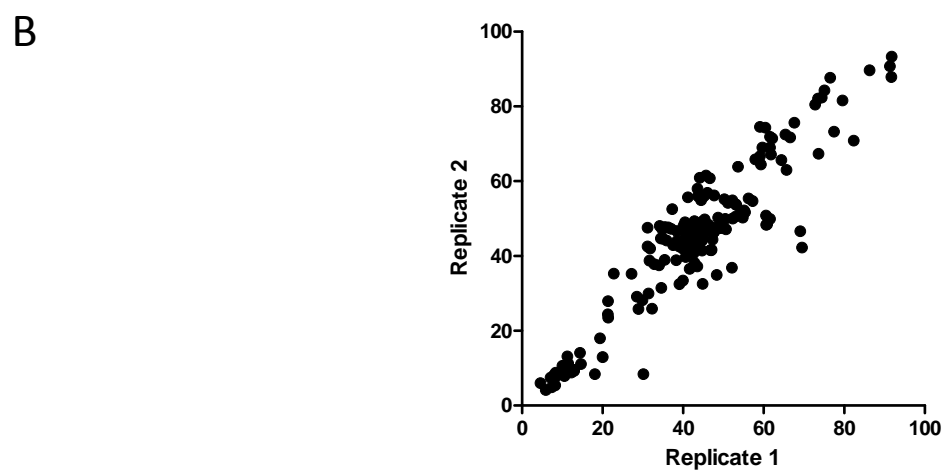

Supplement: Additional file 2 — Figure S2: Quality control on methylation assays. (a) Prior to bisulphite treatment, unmethylated and methylated DNA were mixed together in the ratios described. (i) ZAC differentially methylated region (DMR); (ii) GRB10 germ-line DMR; iii: GNAS germ-line DMR; (iv) MCTS2 DMR; (v) KvDMR; (vi) SNRPN DMR. (b) Reproducibility of experiments. The same DNA was independently bisulphite converted and the pyrosquencing assay run. Individual C-phosphate guanines (CpGs) for replicate 1 were plotted against replicate 2. The r2 of the correlation was 0.86 and the gradient of the trend-line 0.99. When this is plotted per DMR, r2 is 0.96 and x = 1.05. Limits of agreement calculated by a Bland-Altman correlation show a difference of 1.60 between the two replicates. This is not significant. [file 1756-8935-4-1-S2.PDF]

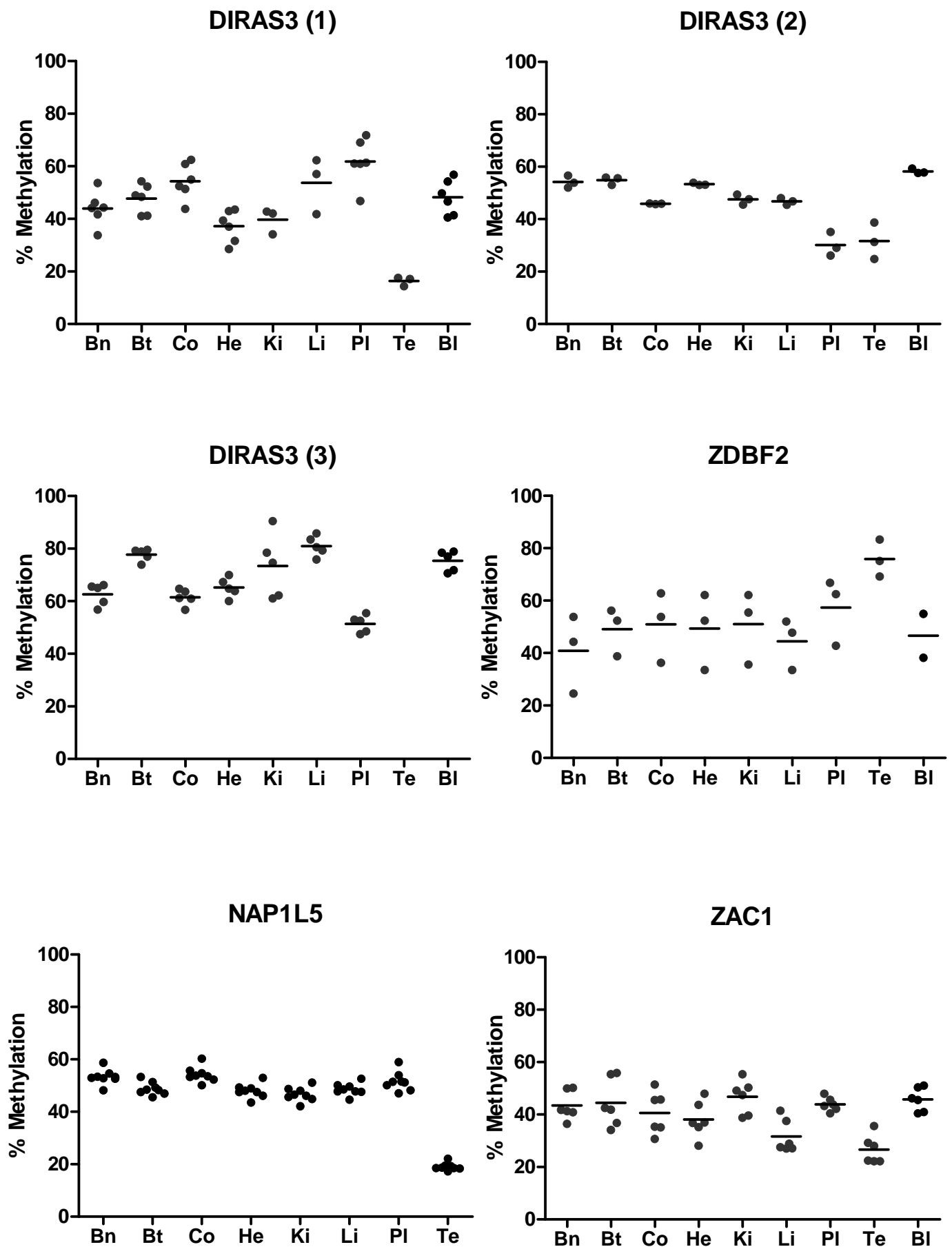

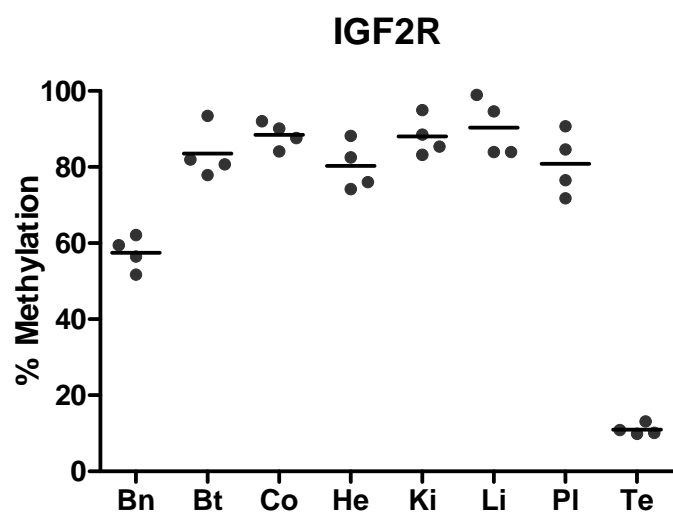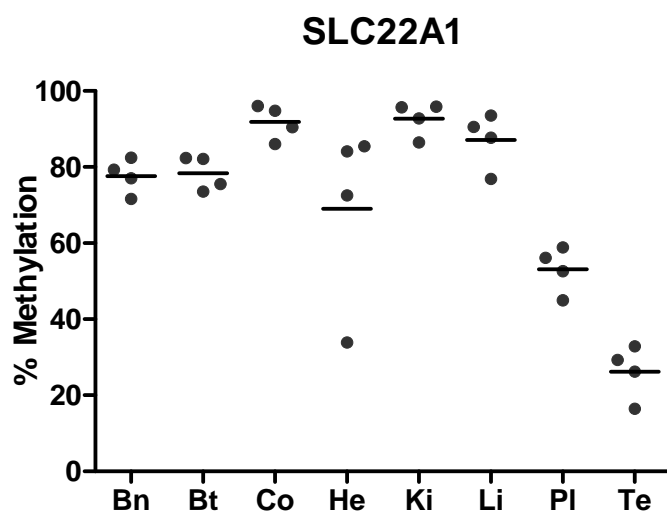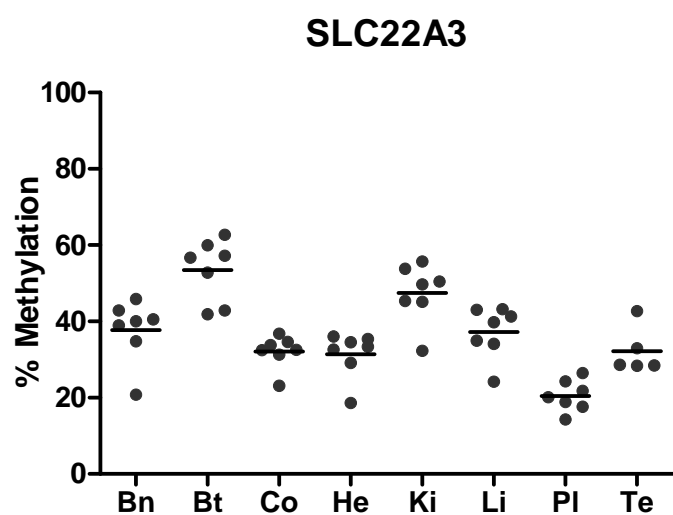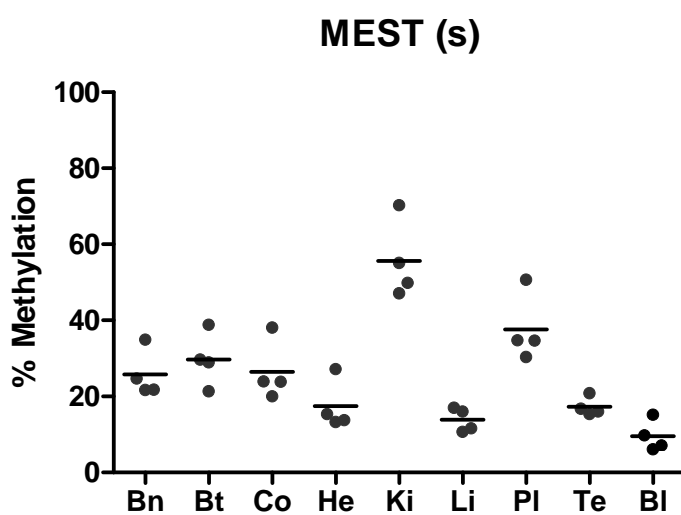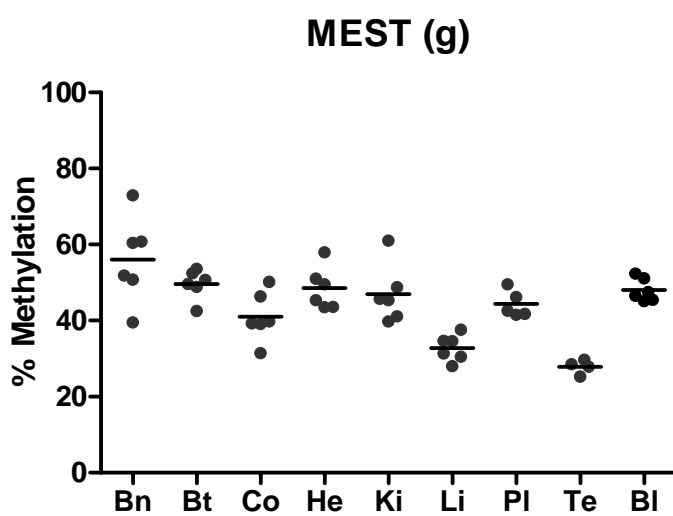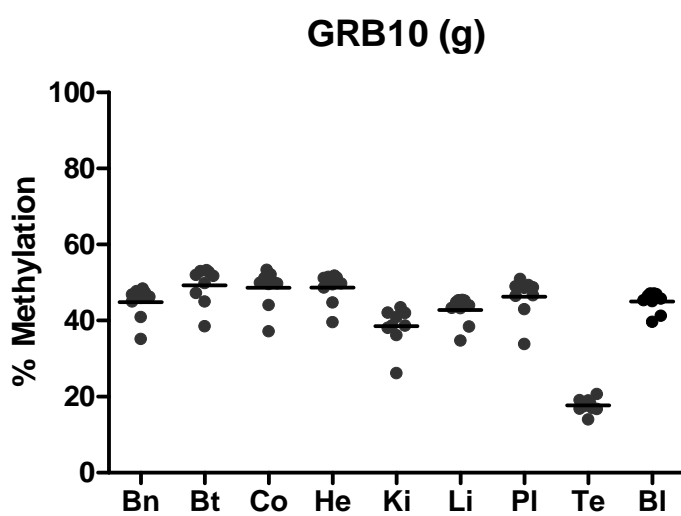

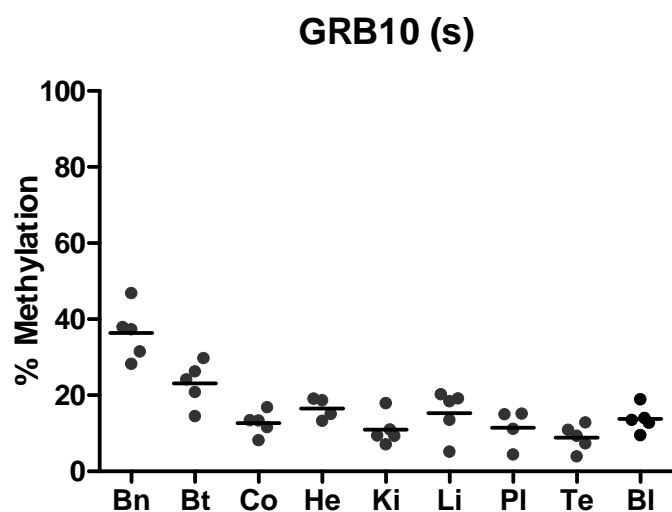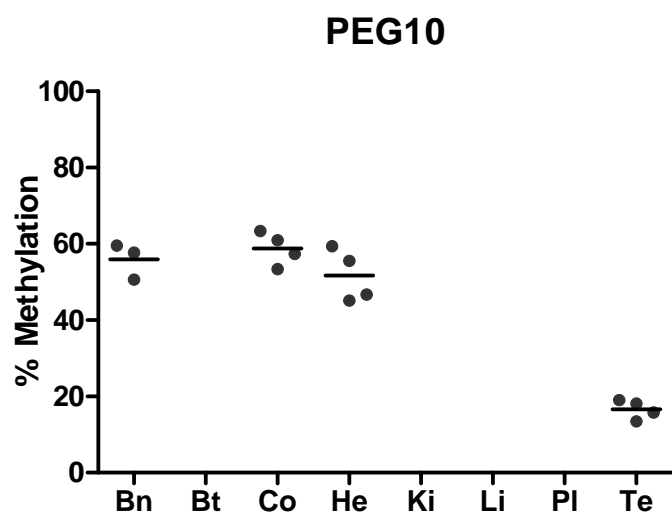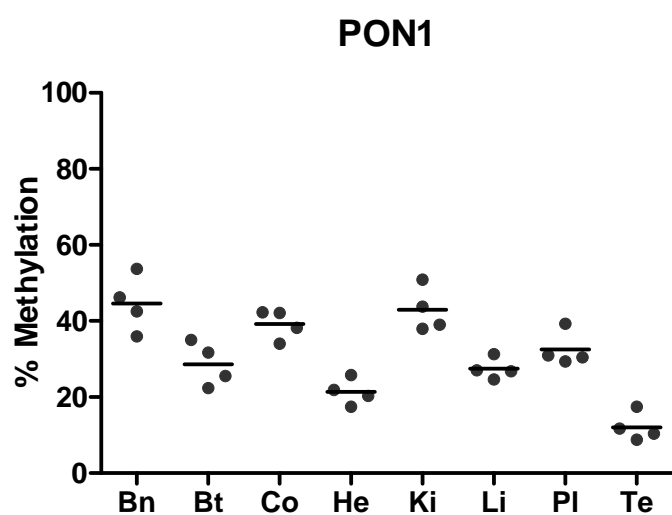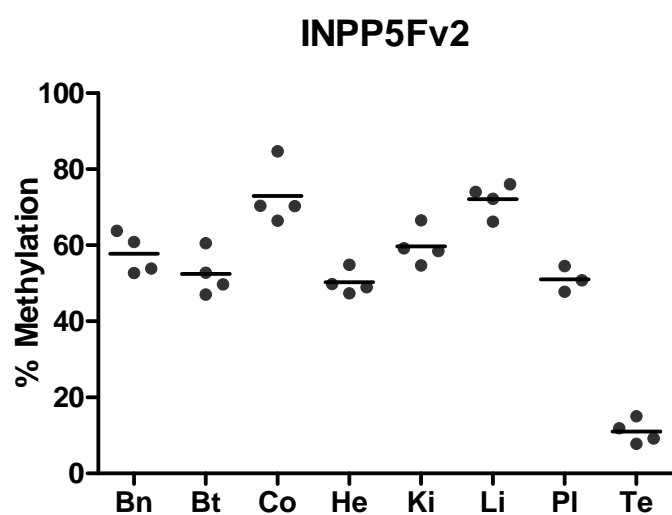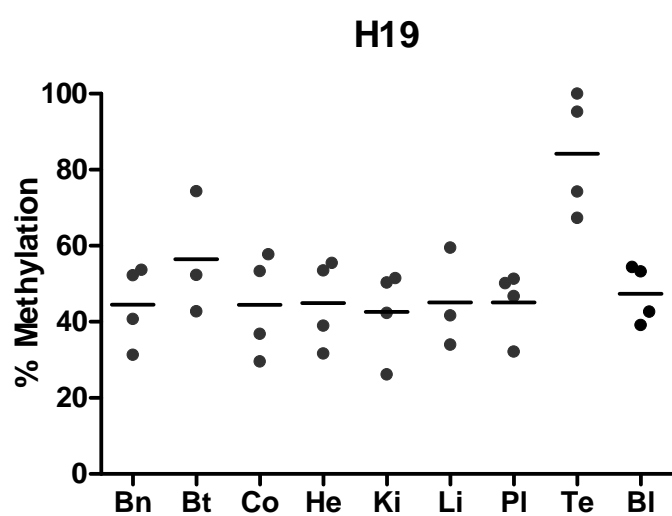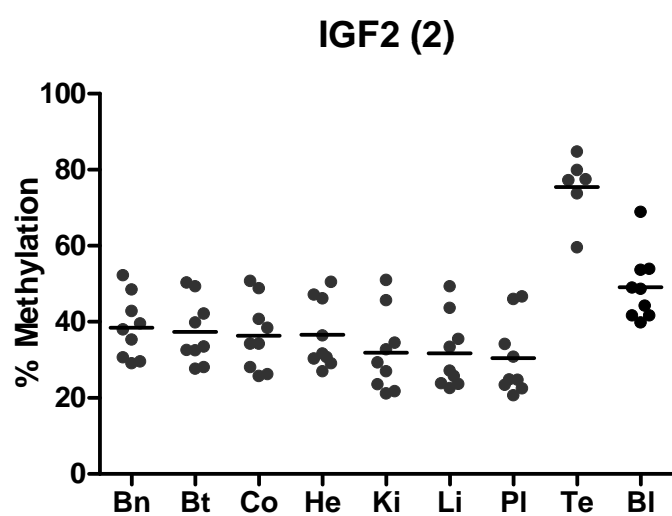

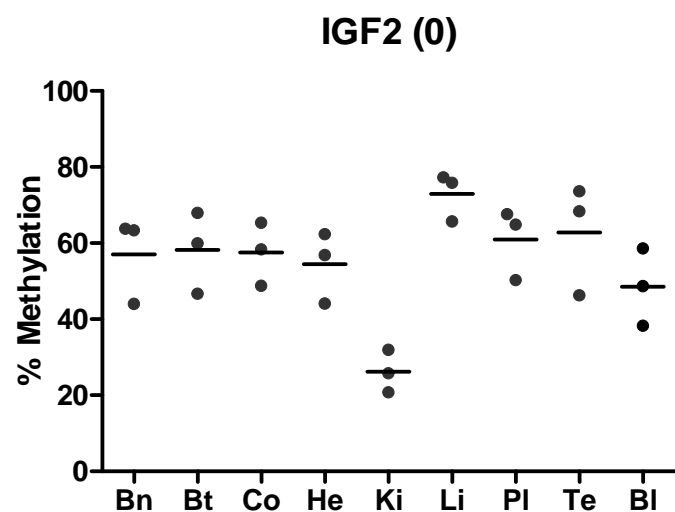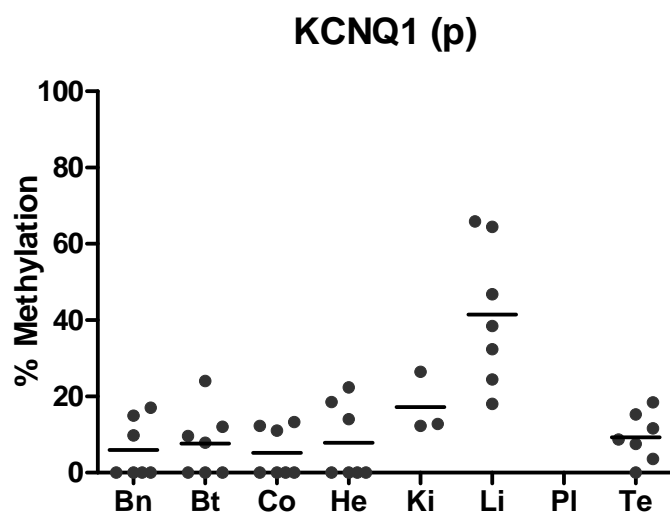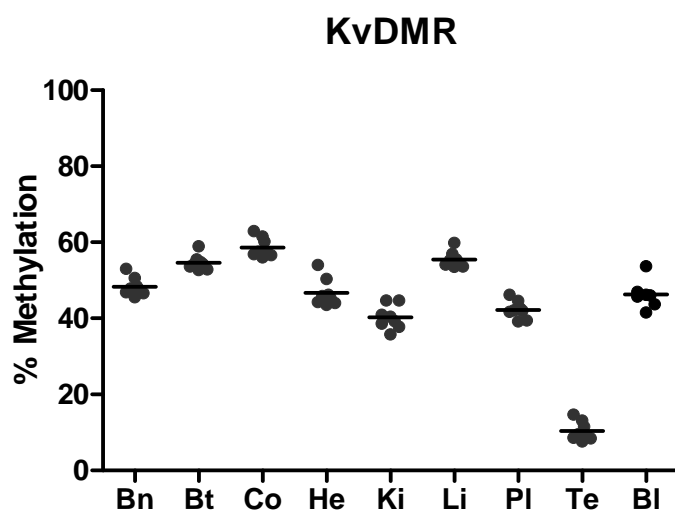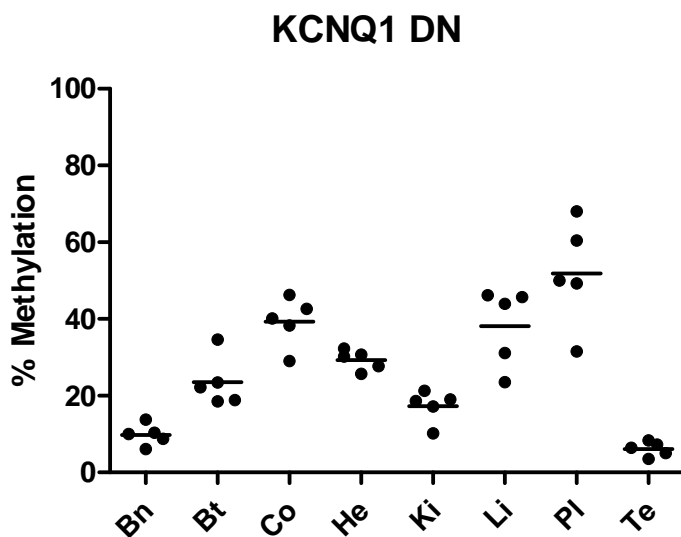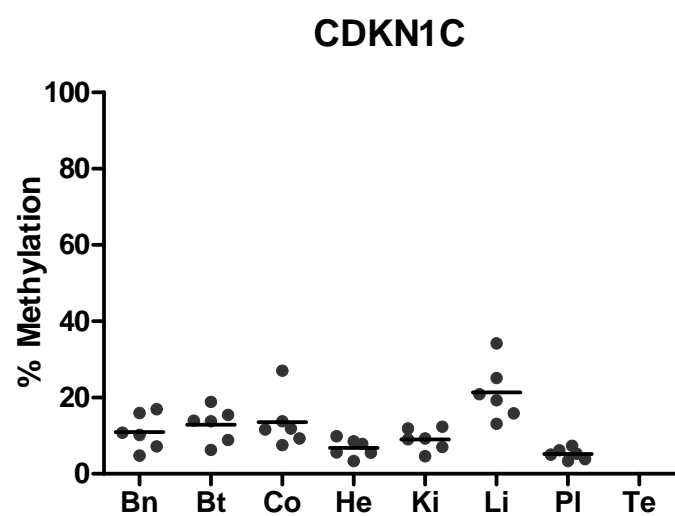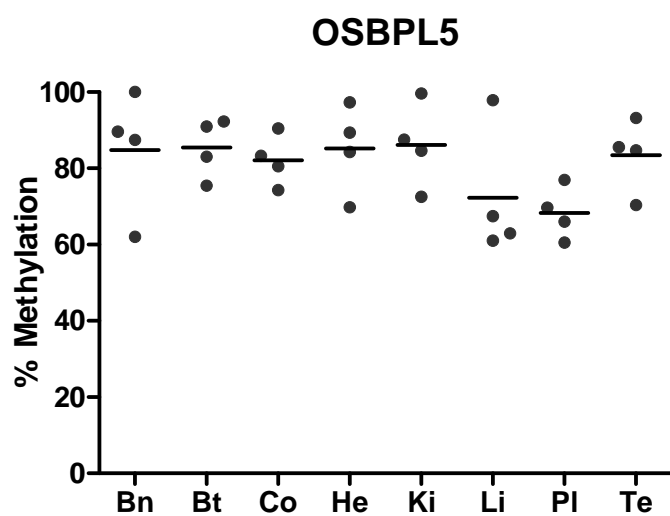

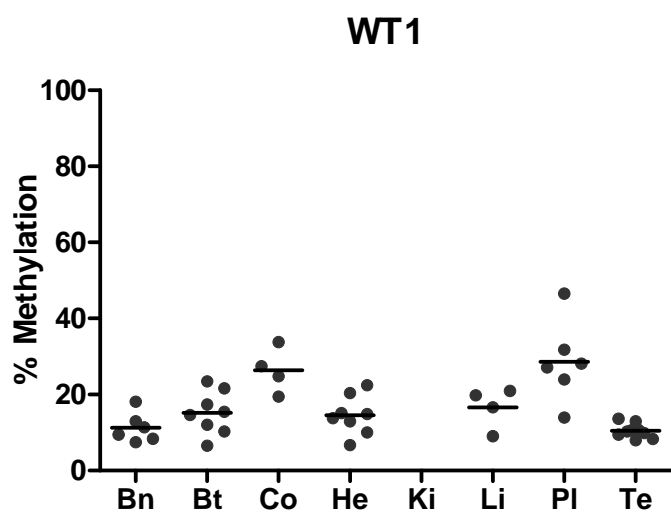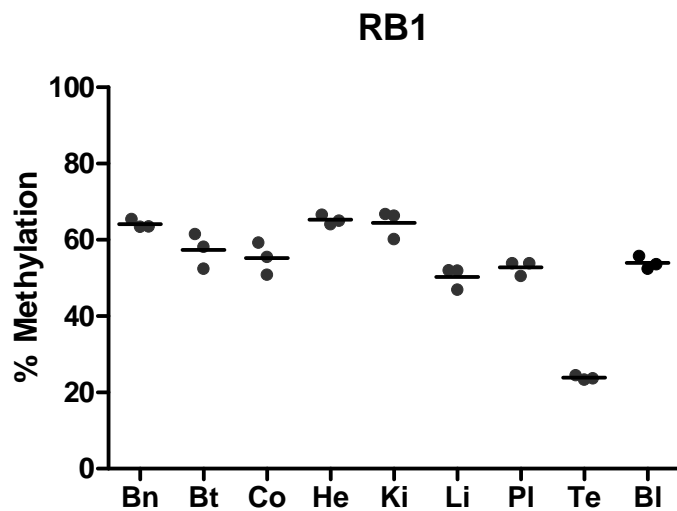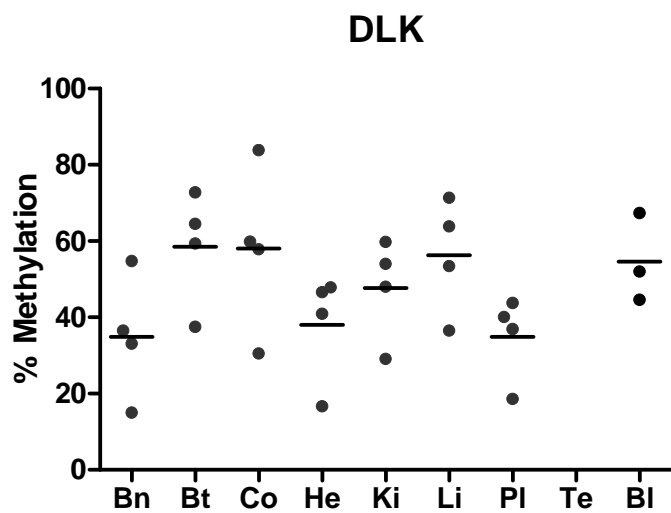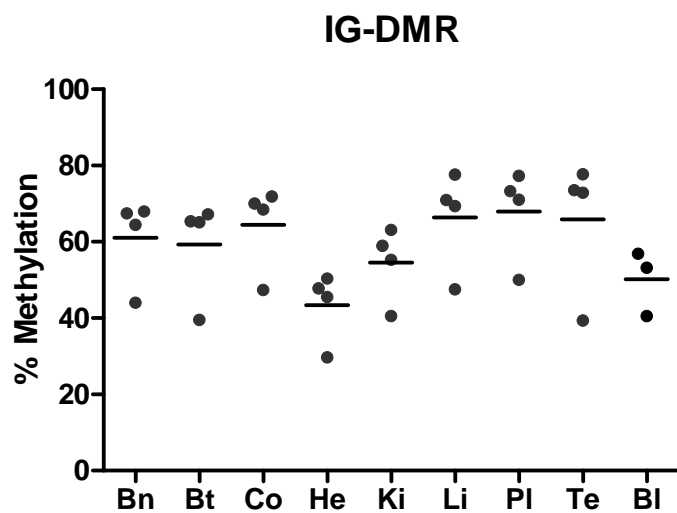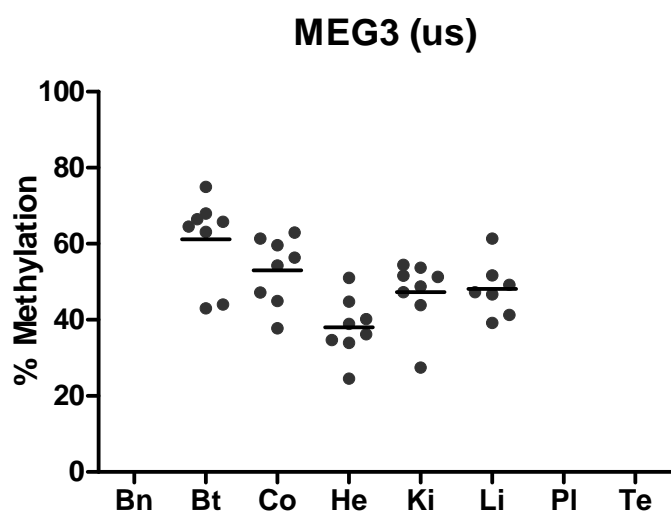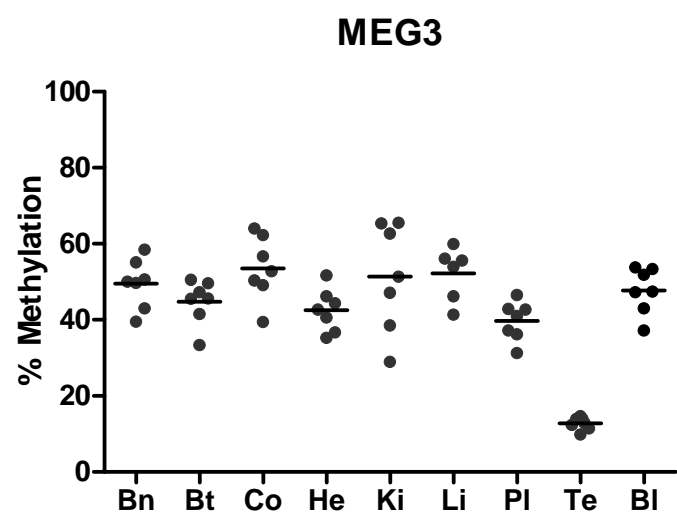

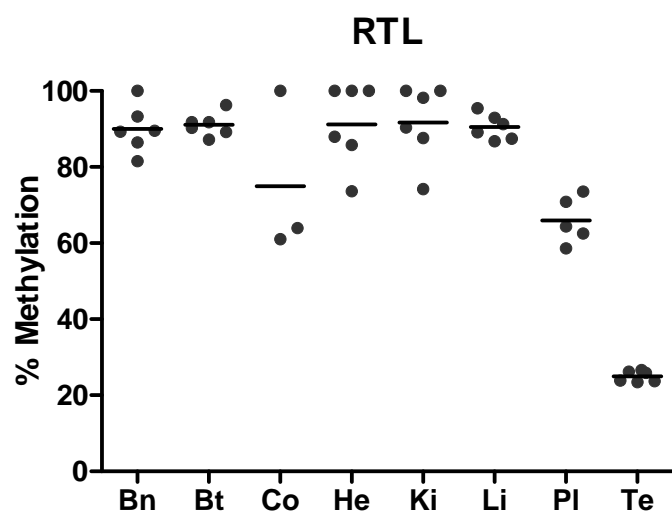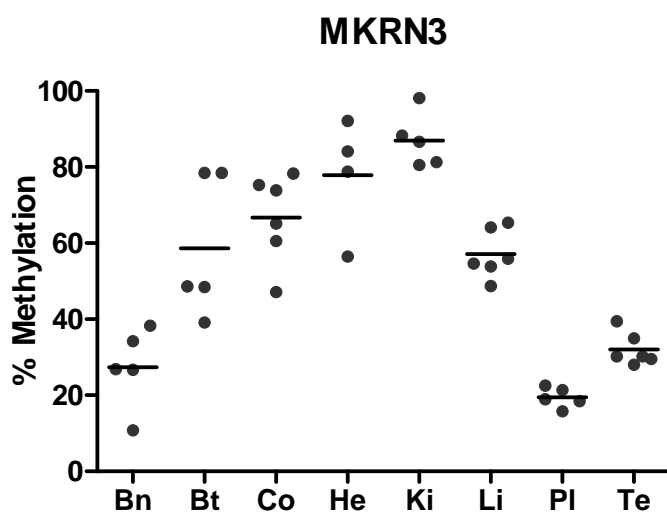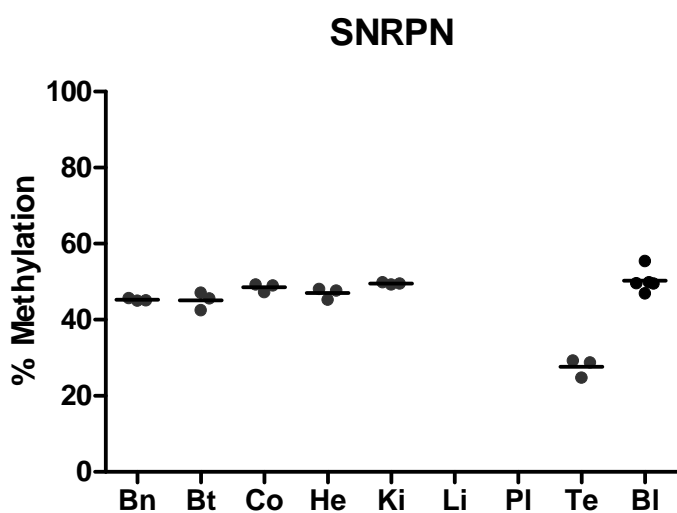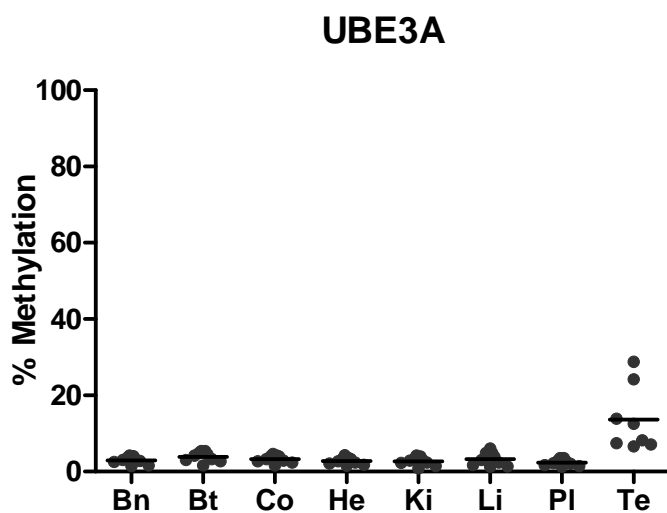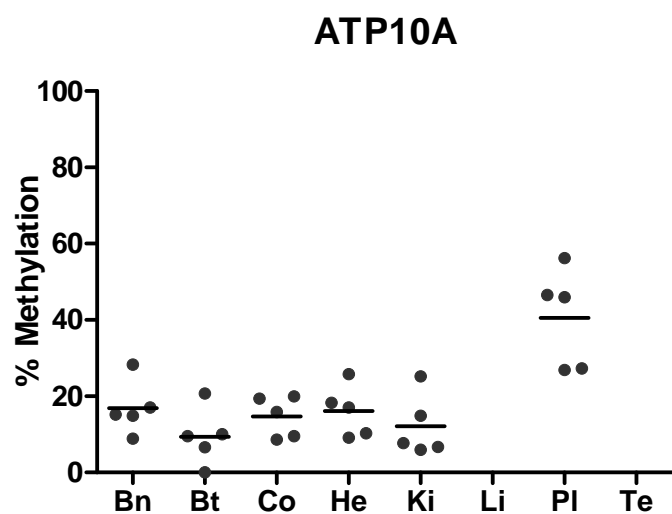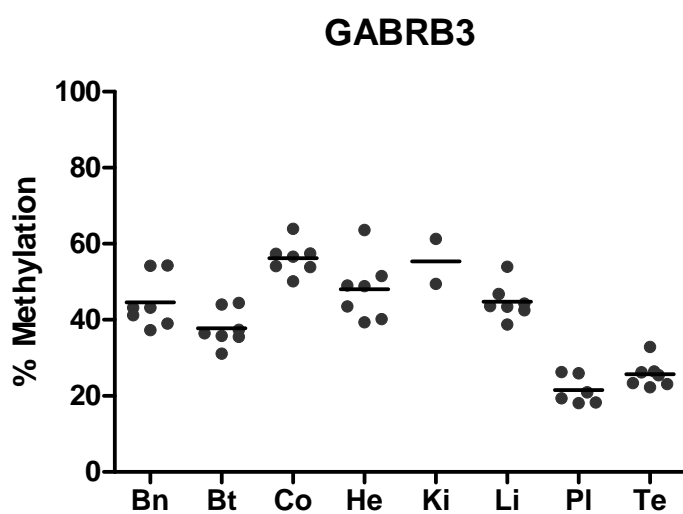

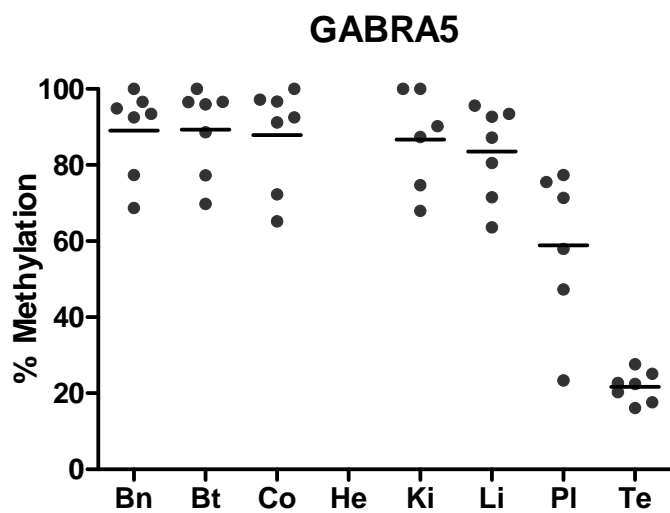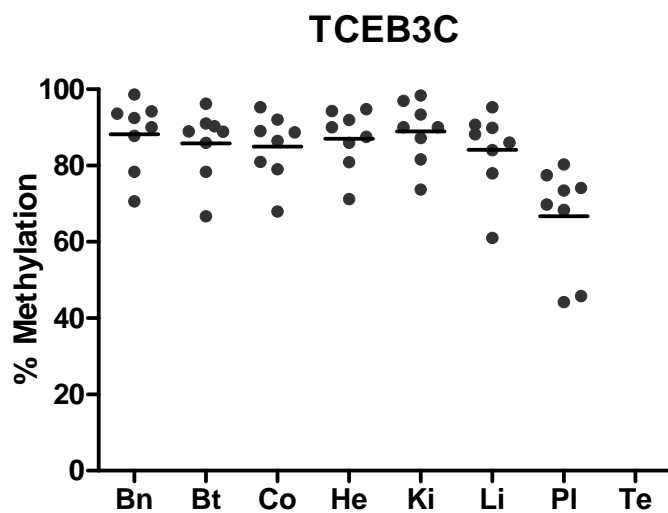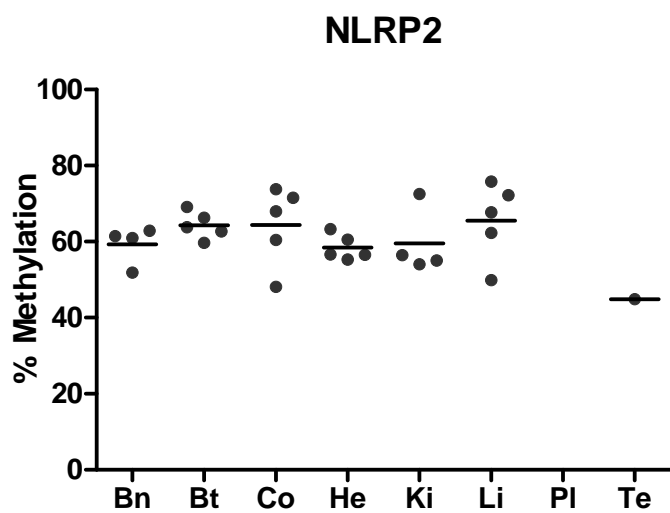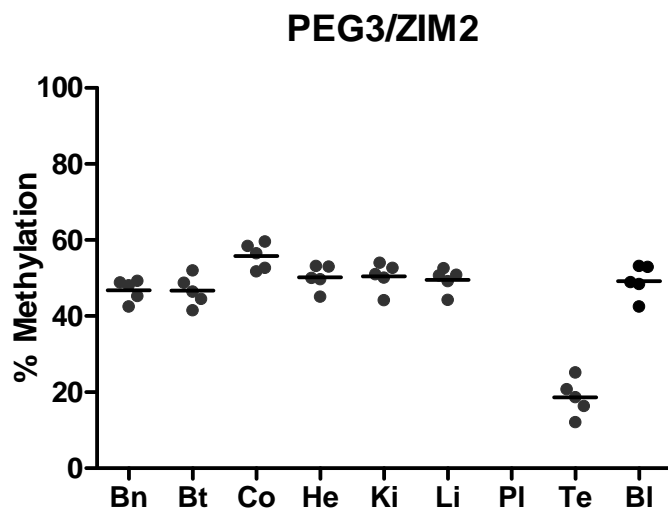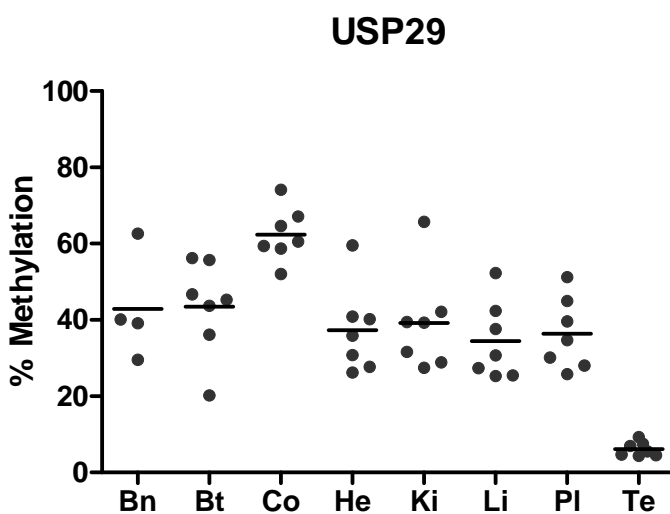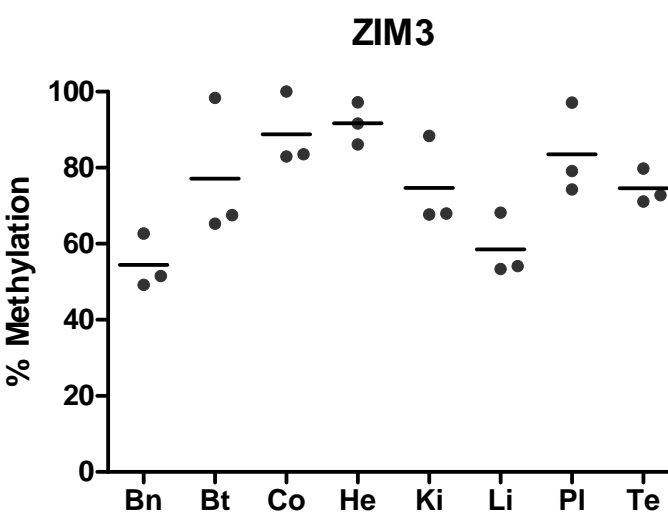

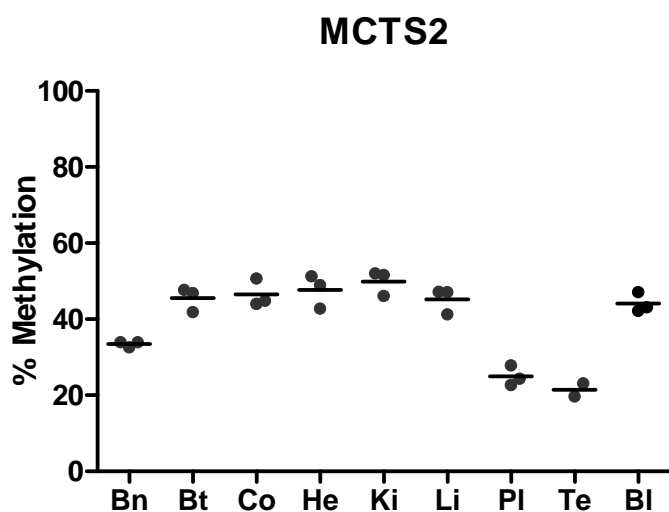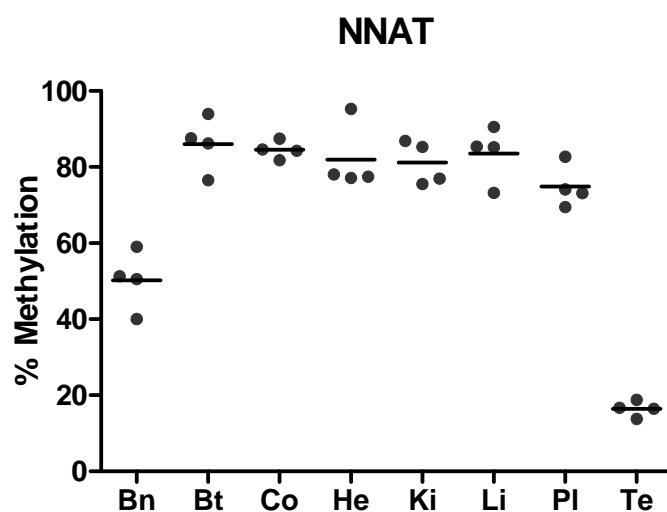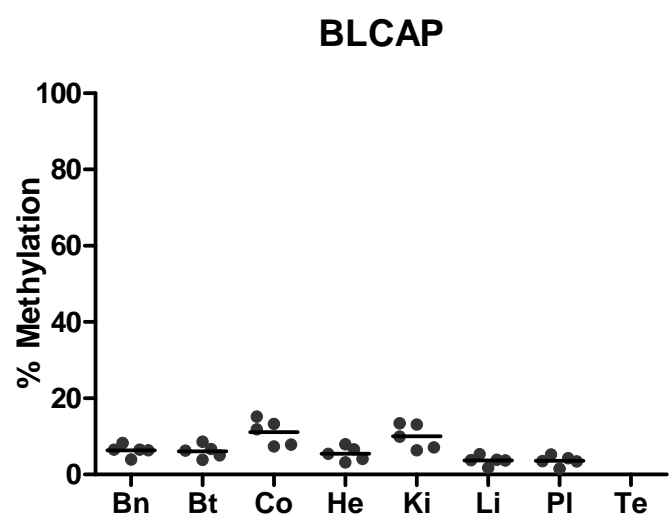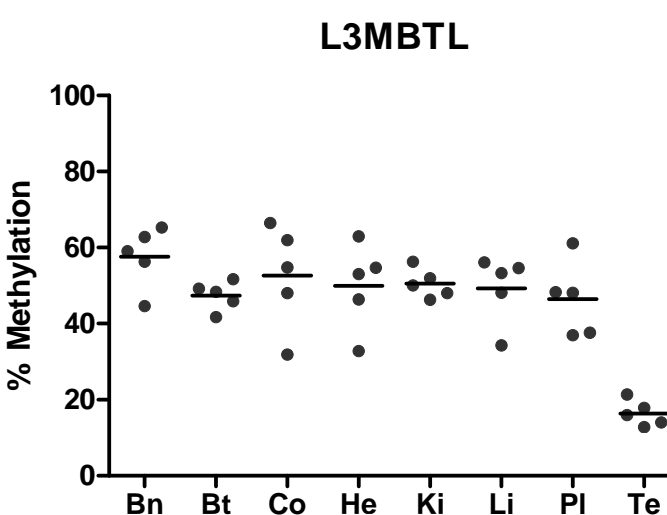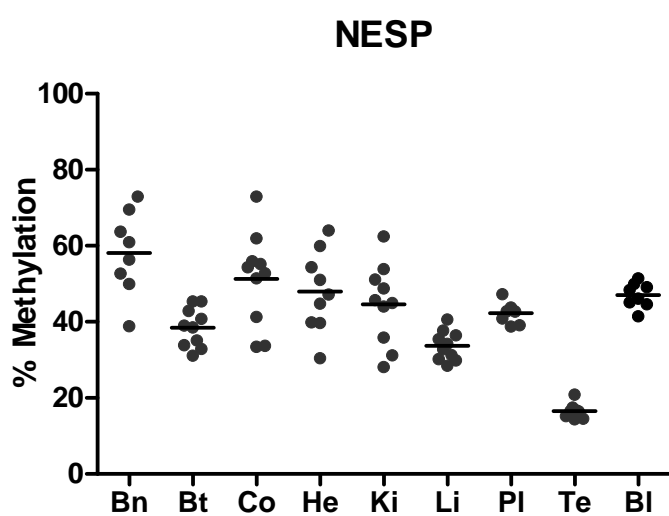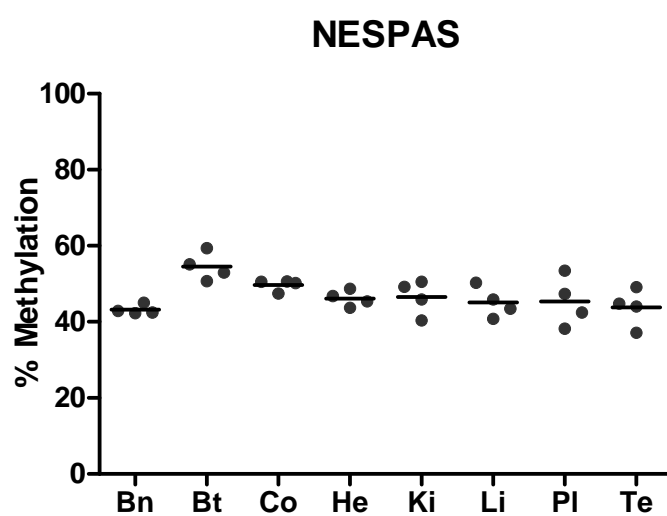

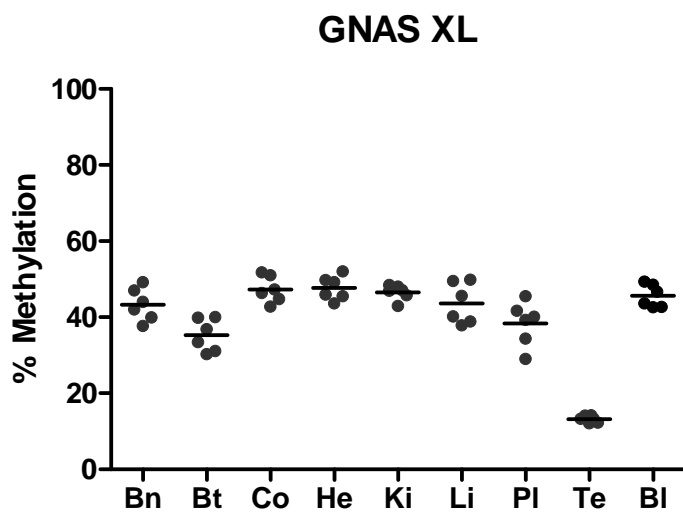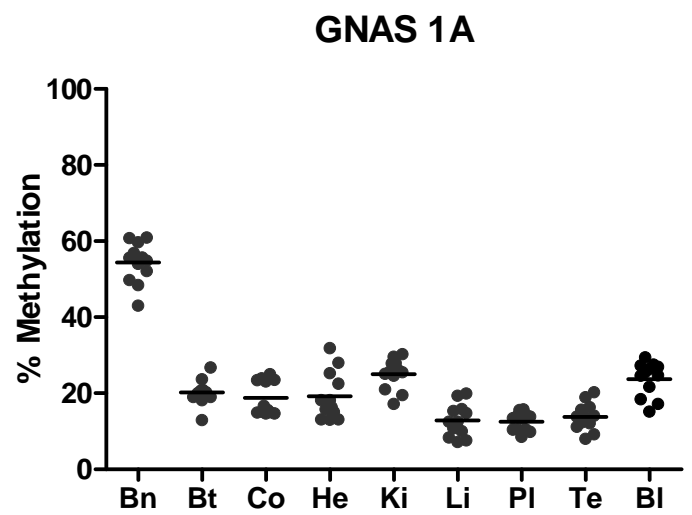

Supplement: Additional file 3 — Figure S2: Methylation levels at eight different adult tissues for 50 regions assayed (in chromosome order). Bn = brain; Bt = breast; Co = colon; He = heart; Ki = kidney; Li = liver; Pl = placenta; Te = testis; Bl = blood. Each data point represents an individual C-phosphate guanine (CpG). Bars represent the mean methylation level. [file 1756-8935-4-1-S3.PDF]

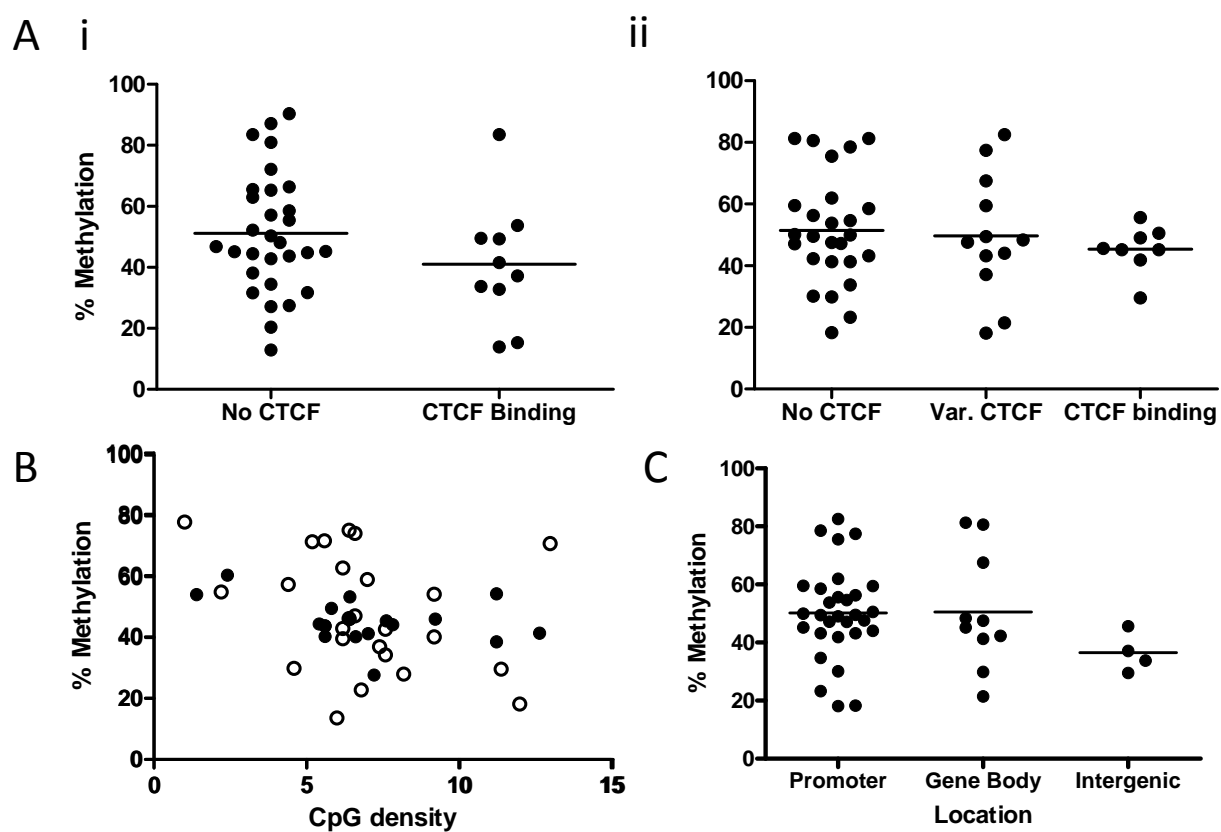

Supplement: Additional file 4 — Figure S3: Comparison of region characteristics with methylation levels. (a) Effect of CTCF binding on methylation levels. (i) Average methylation levels of differentially methylated region (DMR) assays in all tissues (CTCF binding determined from Encode Data on UCSC database). (ii) Methylation levels of DMR assays in liver. (b) Correlation between C-phosphate guanine (CpG) densities of each assay with average somatic methylation level reported. Closed circles = germ-line DMRs; open circles = somatic DMRs. (c) Effect of genomic position of DMR on methylation levels. [file 1756-8935-4-1-S4.PDF]

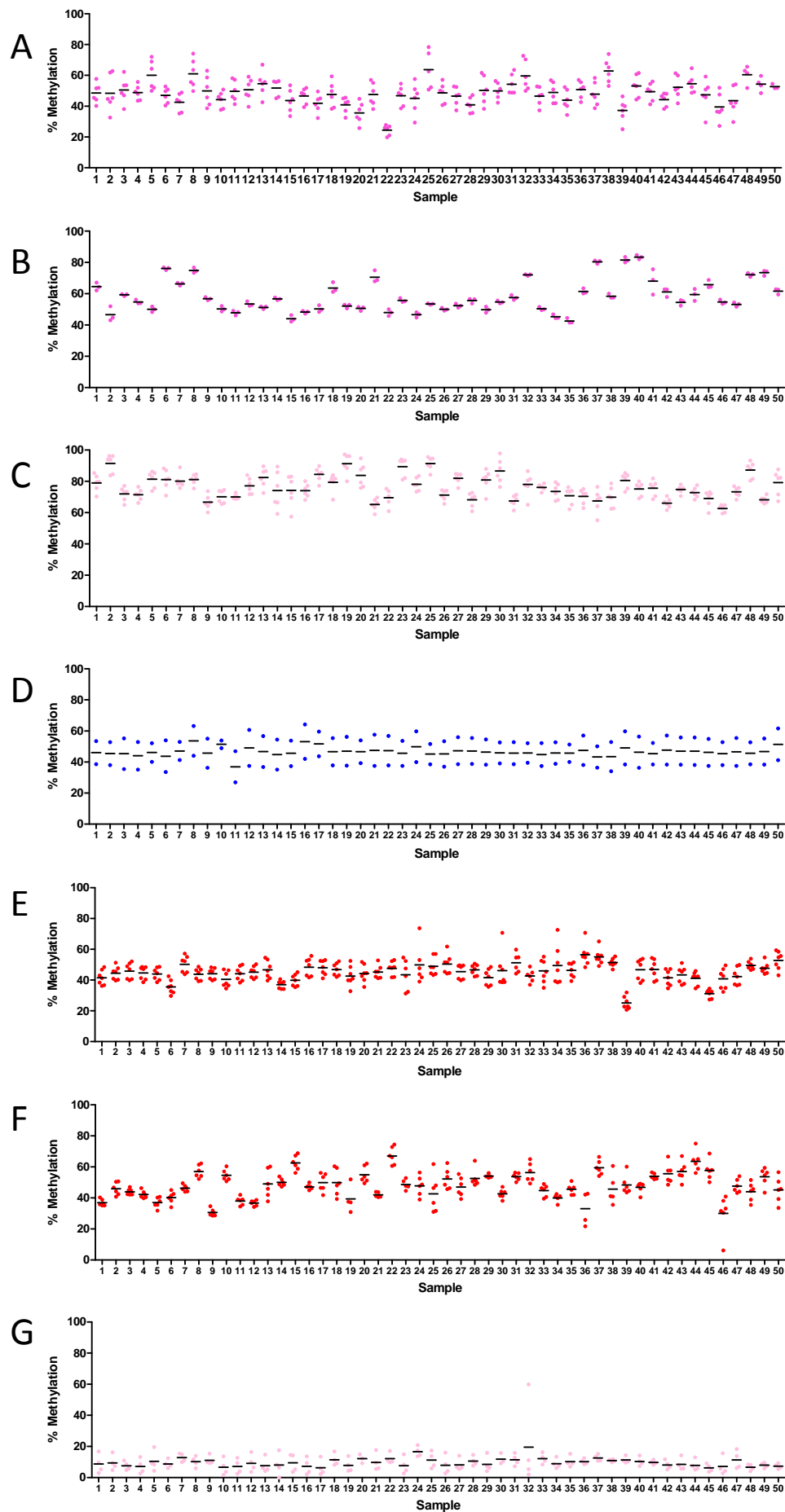

Supplementary Figure 4A

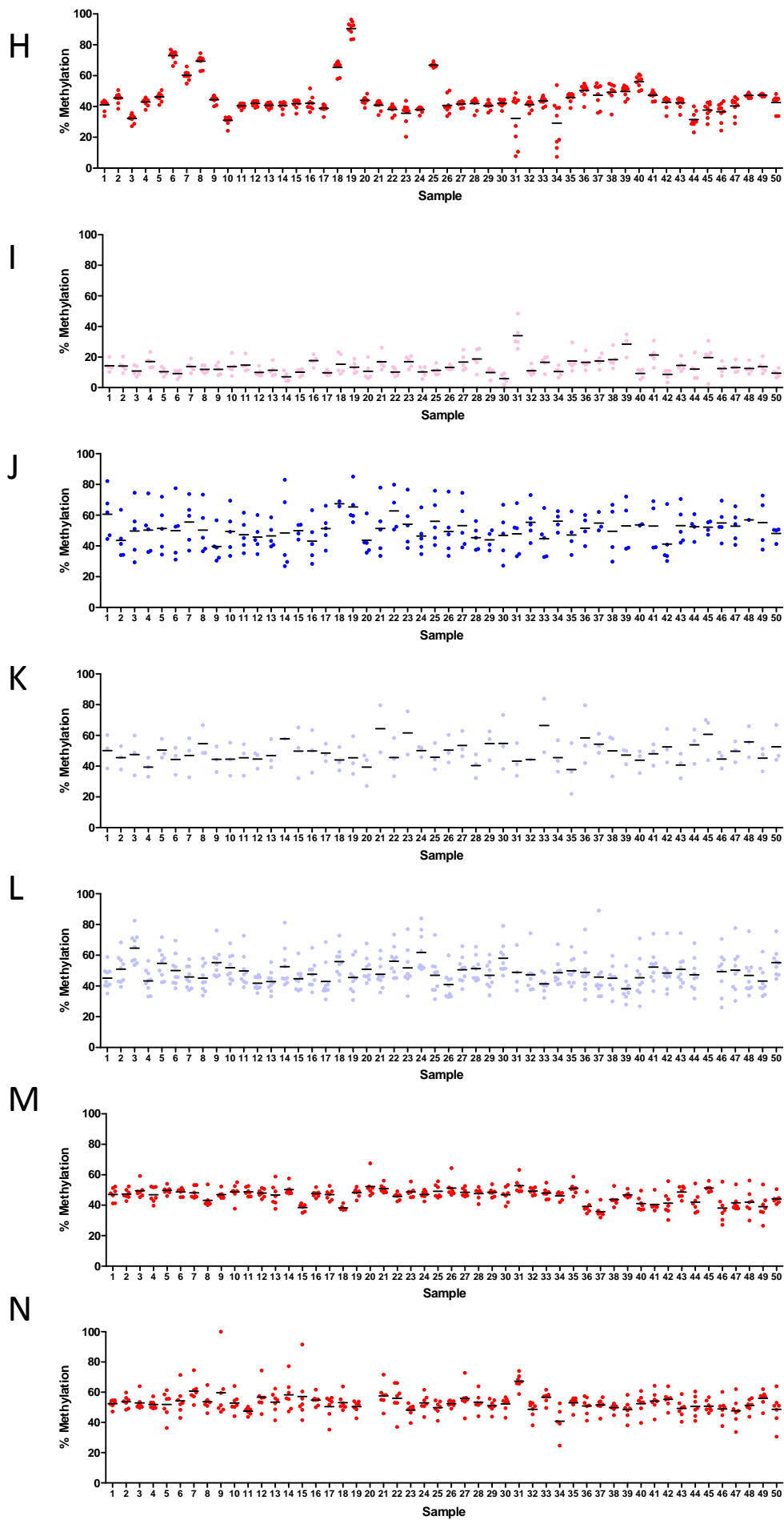

Supplementary Figure 4B

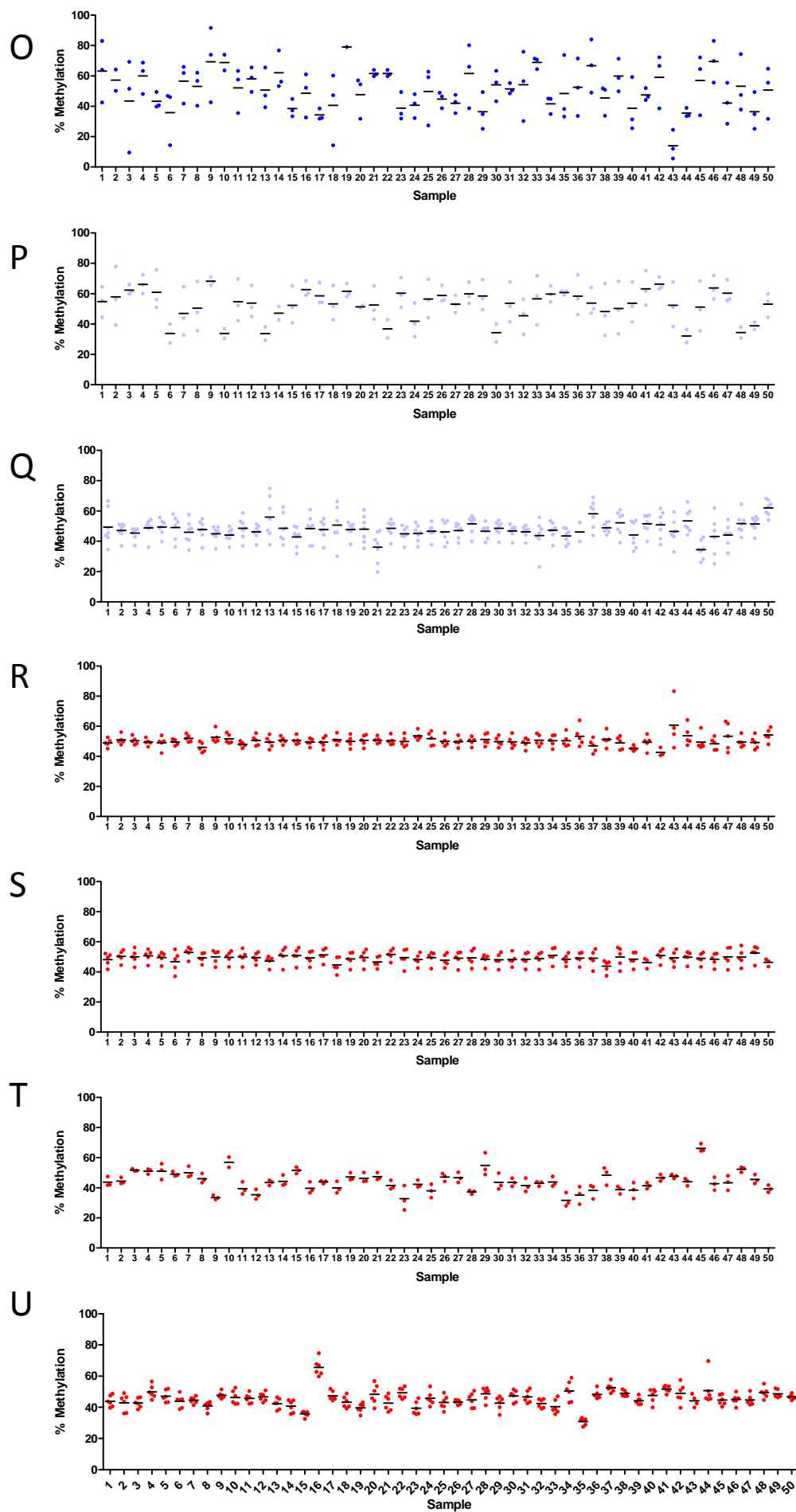

Supplementary Figure 4C

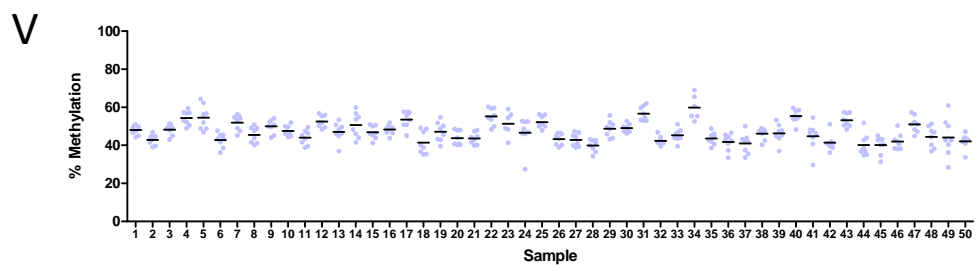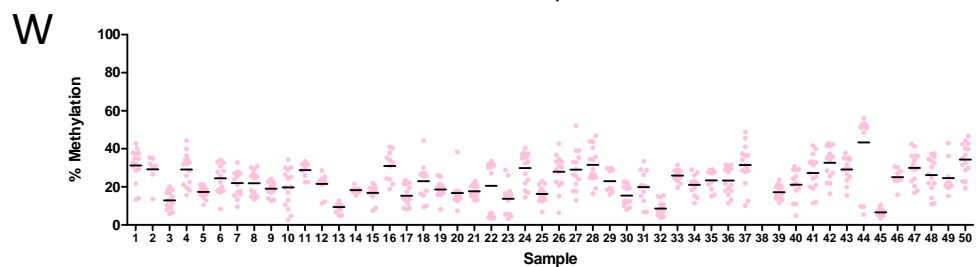

Supplement: Additional file 5 — Figure S4: Methylation levels reported by individual C-phosphate guanines (CpGs) in 50 different individuals at 23 different differentially methylated regions (DMRs. Red = maternal germ-line DMRs; pink = maternal somatic DMRs; dark blue = paternal germ-line DMRs; light blue = paternal somatic DMRs. Each data point represents an individual CpG. Bars represent the mean methylation levels. A = DIRAS3 (1); B = DIRAS3 (2); C = DIRAS3 (3); D = ZDBF2; E = ZAC; F = MEST (g); G = MEST (s); H = GRB10 (g); I = GRB10 (s); J = H19; K = IGF2-0; L = IGF2-2; M = KvDMR; N = RB1; O = DLK; P = IG-DMR; Q = MEG; R = SNRPN; S = PEG3; T = MCTS2; U = NESP; V = GNAS XL; W = GNAS 1A. [file 1756-8935-4-1-S5.PDF]

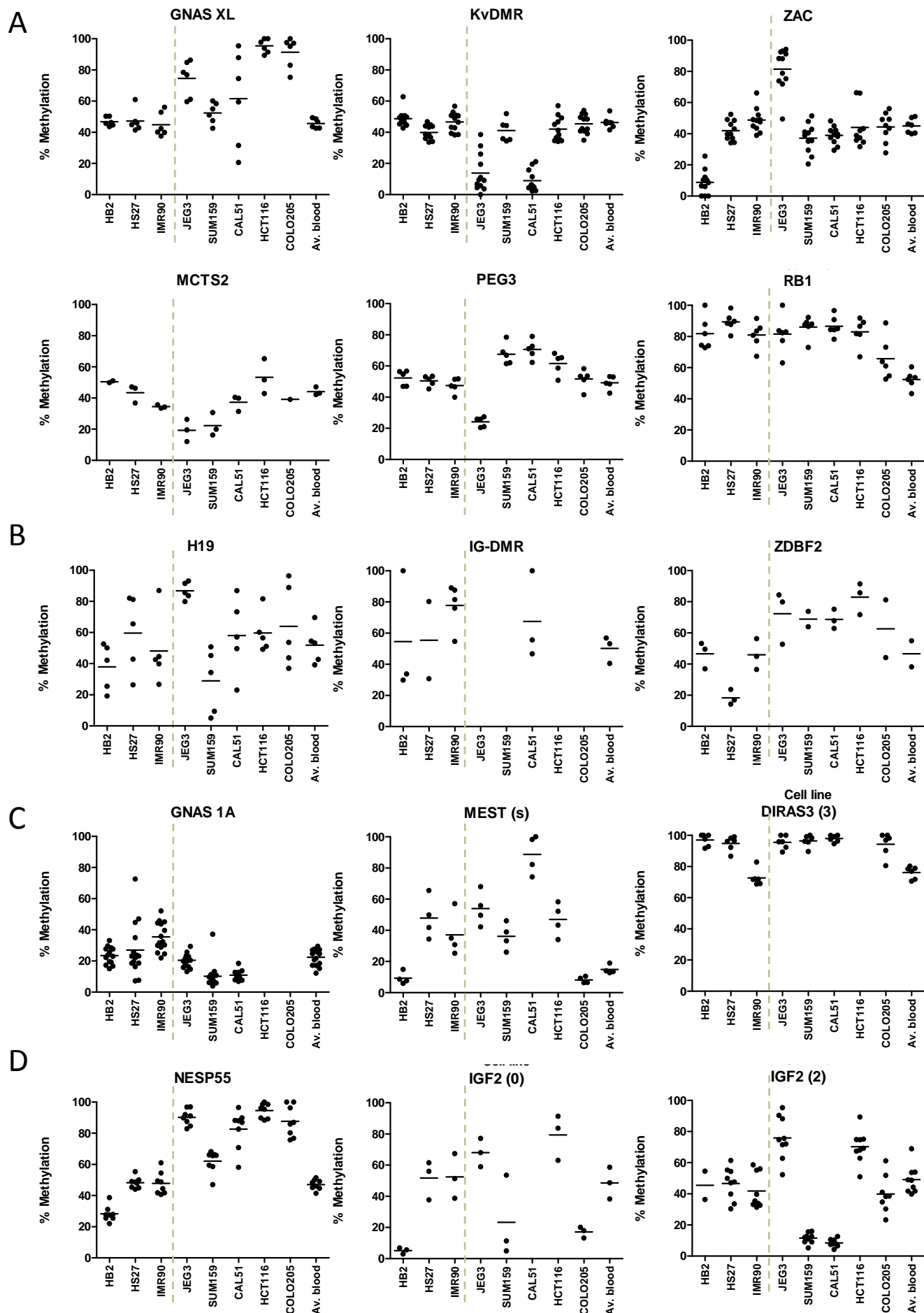

Supplement: Additional file 6 — Figure S5: Methylation levels in eight different cell lines and the average blood methylation levels. Cell lines to the left of the dashed line are normal, whereas cell lines to the right of the line are derived from cancerous samples. Each data point represents an individual C-phosphate guanine (CpG). (a) Maternal germ-line differentially methylated regions (DMRs). (b) Paternal germ-line DMRs. C = maternal somatic DMRs; D = maternal somatic DMRs. [file 1756-8935-4-1-S6.PDF]
